# Dietary patterns, malnutrition, muscle loss and sarcopenia in cancer survivors: findings from the UK Biobank

**DOI:** 10.1007/s11764-023-01428-8

**Published:** 2023-07-20

**Authors:** Annie R Curtis, Katherine M Livingstone, Robin M Daly, Barbara Brayner, Gavin Abbott, Nicole Kiss

**Affiliations:** 1 Institute for Physical Activity and Nutrition, Deakin University, Geelong, Australia; 2 Allied Health Research, Peter MacCallum Cancer Centre, Melbourne, Australia

**Keywords:** Dietary patterns, Malnutrition, Muscle mass, Sarcopenia, Cancer

## Abstract

**Purpose:**

To identify dietary patterns derived from protein, polyunsaturated fatty acids (PUFA) and vitamin D and examine associations with malnutrition, low muscle mass and sarcopenia in cancer survivors.

**Methods:**

This cross-sectional study included cancer survivors (*n* = 2415) from the UK Biobank (age [mean ± SD] 59.7 ± 7.1 years; 60.7% female). The Oxford WebQ 24-h dietary assessment estimated food and nutrient intakes. Reduced rank regression derived dietary patterns (response variables: protein [g/kg/day], PUFA [g/day] and vitamin D [μg/day]). Adjusted logistic regression analysis examined associations between dietary patterns and malnutrition, low muscle mass and sarcopenia.

**Results:**

Three dietary patterns were identified: (i) ‘high oily fish and nuts’, characterised by higher oily fish and nuts and seeds intake; (ii) ‘low oily fish’, characterised by lower oily fish intake and higher potato intake; and (iii) ‘meat and dairy’, characterised by higher intake of meat, poultry and dairy. Eighteen percent of participants were malnourished, 5% had low muscle mass and 6.5% had sarcopenia. Odds of being malnourished were significantly lower with adherence to a ‘high oily fish and nuts’ pattern (OR: 0.57; 95% CI: 0.50, 0.65) and ‘low oily fish’ pattern (OR: 0.81; 95% CI: 0.73, 0.90). The ‘meat and dairy’ pattern was not associated with malnutrition. No dietary patterns were associated with low muscle mass or sarcopenia.

**Conclusions:**

Energy-rich dietary patterns were associated with lower odds of malnutrition in cancer survivors but did not influence muscle mass or sarcopenia risk.

**Implications for Cancer Survivors:**

Better understanding of dietary patterns may improve cancer-related outcomes for cancer survivors.

**Supplementary Information:**

The online version contains supplementary material available at 10.1007/s11764-023-01428-8.

## Introduction

People with cancer are at high risk of developing malnutrition, low muscle mass and sarcopenia due to adverse tumour or treatment-related side effects [1], all of which are associated with morbidity and mortality, poor quality of life (QOL) and worse treatment tolerance [2, 3]. Importantly, the risk of malnutrition and muscle loss may remain for long after the completion of treatment due to the presence of ongoing treatment-related toxicities.

Dietary intake is regarded as a key modifiable lifestyle factor for managing cancer-related malnutrition, low muscle mass and sarcopenia, with current interventions focussing on adequate energy intake and key nutrients such as protein, omega-3 fatty acids and vitamin D [4, 5]. In cancer, the benefits of omega-3 fatty acids and vitamin D supplementation for weight maintenance and maintenance of muscle mass and strength have been widely reported, particularly in patients with an existing deficiency [6, 7]. However, other studies have reported no associations between these nutrient intakes and body composition or important outcomes such as QOL [8, 9]. One potential reason for this disparity is that foods and nutrients are not consumed in isolation and are instead interconnected components of dietary patterns.

Dietary patterns reflect the quantity, variety and groupings of foods and nutrients and their complex synergistic interactions [10]. We previously conducted a scoping review which identified the following: (i) few studies have examined the effects of dietary patterns on malnutrition, low muscle (lean) mass and sarcopenia in people with cancer, and (ii) heterogeneity with regard to cancer types, treatment types and dietary patterns investigated [11]. Reduced rank regression (RRR) is a method used to derive dietary patterns based on a priori knowledge of nutrients (or biomarkers) relevant to the outcomes under investigation [12]. These novel dietary patterns can build upon existing nutrient-focussed literature by identifying the optimal food combinations that may be effective for preventing and treating malnutrition, low muscle mass and sarcopenia. However, no studies have employed this approach in the investigation of dietary intake on nutritional status or body composition in cancer survivors. The aim of this study was to investigate the cross-sectional associations between dietary patterns; derived based on intake of protein; polyunsaturated fatty acids (PUFA) and vitamin D; and the risk of malnutrition, low muscle mass and sarcopenia in cancer survivors.

## Methods

### Study design and participants

The UK Biobank is a population-based cohort study designed to investigate the role of genetic, lifestyle and environmental factors in health and disease [13]. In brief, approximately 500,000 adults aged 40 to 69 years were recruited between 2006 and 2010, and attended one of 22 local assessment centres across England, Scotland and Wales for baseline assessments. Sociodemographic characteristics; lifestyle; health-related data; and physical, anthropometric and biological measurements were collected. In 2009, a 24-h dietary assessment tool, the Oxford WebQ, was introduced to collect dietary information.

### Inclusion criteria

Participants were included in the present analysis if they (i) were diagnosed with cancer (excluding non-melanoma skin) prior to the baseline assessment; (ii) completed the Oxford WebQ dietary assessment at the baseline assessment; (iii) had complete data for exposures, outcomes and covariates and; (iv) consumed ‘plausible’ energy intakes (within the upper-limit of 14,700 kJ/day for women and 17,640 kJ/day for men) [14]. Regarding the latter, energy intakes of 2100 to 14,700 kJ/day (women) and 3360 to 17,640 kJ/day (men) are considered plausible in healthy adult populations [14]. Energy intakes below these ranges were considered clinically plausible in the present study due to the potential for unfavourable nutrition-related side effects of cancer. To acknowledge the impact of a recent cancer diagnosis on nutritional intake, a sub-analysis was performed based on participants that (i) met the above inclusion criteria and (ii) were diagnosed with cancer within 2 years prior to the baseline assessment.

### Study measures

#### Cancer

Cancer type and diagnosis date were retrieved via linkages to national cancer registries. Cancer types were grouped according to International Classification of Diseases (ICD-10) codes (Table S1).

#### Dietary intake

The Oxford WebQ was used to collect information about the consumption of 206 foods and 32 beverages during the preceding 24-h [15]. Total energy and nutrient intakes were determined by multiplying the frequency of consumption of each food by a standard portion size and estimated nutrient composition using UK Nutrient Databank food-composition tables for years 2012–2013 and 2013–2014 [16]. The Oxford WebQ has been validated against biomarkers for protein, potassium, total sugar intake and total energy expenditure, performing well compared to a traditional interviewer-administered multi-pass 24-h dietary recall [17]. Dietary intake was estimated from one Oxford WebQ 24-h dietary recall at the baseline assessment for both the full and sub-samples. One (rather than multiple) dietary recall was used to allow the analysis of dietary intake at the baseline assessment, corresponding with timing of the assessment of malnutrition, low muscle mass and sarcopenia. Alternatively, the use of multiple dietary recalls may have introduced reverse causality bias, where at least one dietary exposure point would have proceeded the assessment of outcome measures. Given the potentially rapid nature by which nutritional status can change, especially after cancer diagnosis [18, 19], the increased exposure period introduced by use of multiple dietary recalls (>1 year) may not have been reflective of dietary intake at the time when malnutrition, low muscle mass or sarcopenia were present.

#### Dietary patterns

Dietary patterns were derived from Oxford WebQ dietary data using RRR analysis. RRR is a statistical technique used to derive dietary patterns based on food group predictor variables and nutrient intake response variables [12]. Foods and beverages were grouped into 41 food groups (Table S2) which reflected categories from the UK National Diet and Nutrition Survey [20]. Food groups were then modified according to their nutrient profiles in line with our chosen nutrient response variables. Nutrient response variables were protein (g/kg/day), PUFA (g/day) and vitamin D (μg/day), in line with established evidence for their favourable effects on nutritional status, muscle mass and/or sarcopenia in cancer [4, 6, 21]. Protein density (g/kg) was calculated by dividing total protein intake (grams) by participant body weight (kg) at the baseline assessment, reflecting the reporting of the current clinical recommendations for protein intake in cancer [4]. The number of RRR-derived dietary patterns corresponds to the number of nutrient response variables. To determine whether the dietary patterns were suitable for further analysis, a cut-off of < 10% for explained variation in response variable intakes was applied based on previous literature [22].

#### Low muscle mass

Low appendicular lean soft tissue (ALST) is reported as low muscle mass herein. Appendicular fat free mass (AFFM) was estimated using bioimpedance analysis (Tanita BC418MA body composition analyser). ALST was estimated using the equation: ALST (kg) = (0.958 × [AFFM (kg)]) − (0.166 × S) − 0.308, where *S* was allocated the value of 0 (females) or 1 (males). This equation was previously developed using data from UK Biobank participants (*n* = 4350) who completed dual x-ray absorptiometry body composition scans [23]. UK Biobank-specific cut-points for low ALST adjusted for body mass index (ALST/BMI) were derived using two standard deviations (SD) below the mean for reference participants aged ≤45 years (Table 1), as per previously reported methods [24]. ALST/BMI and ALST/height^2^ are two common methods of adjusting low muscle mass for body size. Low ALST/BMI was chosen for this analysis as it has previously been shown to be more sensitive for identifying malnutrition, overall and in participants with obesity, in the UK Biobank compared to height^2^ [25].

**Table 1:** Cut-points for low muscle mass, malnutrition and sarcopenia by sex

Outcome	Criteria	Cut-points	
		Male	Female
Low muscle mass^1^	ALST/BMI	< 0.84	< 0.55
Malnutrition^2^	Weight loss	‘Weight loss compared to one year ago’	
	Low BMI (kg/m^2^)	< 20 if < 70 years or < 22 if > 70 years (mild-moderate) *OR* < 18.5 if < 70 years and < 20 if > 70 years (severe)	
	Reduced muscle mass (ALST/BMI)	< 0.94 (mild-moderate) *OR* < 0.84 (severe)	< 0.64 (mild-moderate) *OR* < 0.55 (severe)
	Reduced food intake (kJ/kg/day)	< 115.5	
	Inflammation (CRP, mg/L)	> 5	
Sarcopenia^3^	Low muscle strength	< 27	< 16
	Low muscle mass (ALST/BMI)	< 0.84	< 0.55
	Low physical performance	‘Slow walking pace’	

Abbreviations: *ALST*, appendicular lean soft tissue; *BMI*, body mass index; *CRP*, C-reactive protein

^1^Low muscle mass: two standard deviations (SD) below the mean for reference participants aged ≤ 45 years

^2^Malnutrition: amended definition according to the Global Leadership Initiative on Malnutrition criteria. Weight loss in response to the touch screen question: ‘compared with one year ago, has your weight changed’. Reduced energy intake (kJ/kg/day) based on < 75% energy recommendations from The European Society for Clinical Nutrition and Metabolism. Presence of inflammation indicated by elevated CRP

^3^Sarcopenia: amended definition according to European Working Group on Sarcopenia in Older People 2019 definition. Low muscle strength was defined as low maximum hand grip strength. Low physical performance indicated by slow walking pace in response to the question ‘how would you describe your usual walking pace?’

#### Malnutrition

Malnutrition was assessed according to the Global Leadership Initiative on Malnutrition (GLIM) criteria [26]. Malnutrition is diagnosed when at least one phenotypic (weight loss, low BMI, reduced muscle mass) and one etiologic criterion (reduced food intake or assimilation, inflammation) are met. Reflecting the availability of data, adaptations were made to the GLIM criteria for the present analysis (Table 1), in line with previous research [27]. Regarding phenotypic criteria, weight loss was indicated by response to the touchscreen question: ‘compared with one year ago, has your weight changed’. BMI was constructed from body weight (kg; Tanita BC-418MA Body Analyzer; Tanita Corporation of America, Inc.) and standing height (Seca 202 device). Reduced muscle mass was estimated based on ALST/BMI as outlined above. Mild-moderate and severe malnutrition are distinguished according to the severity of phenotypic GLIM criteria [26]. Thus, cut-points for low muscle mass were derived based on 1 SD below the mean for healthy individuals, in line with previous literature [28]. Regarding etiological criteria, reduced food intake was indicated when energy intake was < 75% of the mid-point of current recommendations for energy (105–126 kJ/kg/day) [4]. Inflammation was estimated from c-reactive protein (CRP) levels (> 5 mg/L) [29].

#### Sarcopenia

Sarcopenia was defined according to the revised definition by the European Working Group on Sarcopenia in Older People 2019 (EWGSOP2) [30]. The EWGSOP2 definition includes low muscle strength (probable sarcopenia) plus low muscle mass (sarcopenia) and low physical performance (severe sarcopenia) and was adapted to reflect data availability (Table 1). Muscle strength reflected the maximum value for right or left hand grip strength (Jamar J00105 hydraulic hand dynamometer). Low muscle mass was estimated based on ALST/BMI. Low physical performance was indicated by the response to the touchscreen question: ‘how would you describe your usual walking pace?’.

#### Demographic information and potential confounders

Age and sex were self-reported and years since cancer diagnosis were calculated using the date of first cancer diagnosis and date of attending the baseline assessment. Ethnic background was collapsed into ‘prefer not to answer’, ‘Anglo-Saxon’ (White, British, Irish) and ‘other’ due to the majority of participants identifying as ‘White’. Townsend Deprivation Index indicated level of material deprivation and was estimated from national census data. A higher score indicated a greater degree of material deprivation. Smoking status was categorised as ‘never’, ‘previous’ or ‘current’, and those who responded ‘Prefer not to answer’ were excluded. Physical activity was assessed using the International Physical Activity Questionnaire. Metabolic equivalent task scores were calculated from the time spend in specific activities and were grouped (‘low’, ‘medium’, ‘high’) as per previously literature [31]. Overweight (BMI ≥ 25 kg/m^2^) and obesity (BMI ≥ 30 kg/m^2^) were defined according to the World Health Organisation [32].

### Statistical analysis

Complete case analysis was used. Descriptive statistics included mean ± SD for continuous variables and number (%) for categorical variables. Dietary pattern scores were categorised into tertiles for descriptive purposes. For regression models, dietary pattern scores were standardised (*z*-scores) for comparison of effect sizes and treated as continuous variables as per previous studies [33, 34]. Correlation coefficients (interpreted as weak, *r* = 0.1–0.3; moderate, *r* = 0.4–0.6; strong, *r* = 0.7–0.9) were determined between dietary patterns and nutrient response variables. Logistic regression models estimated odds ratios (OR) and 95% confidence intervals (CI) of malnutrition, low muscle mass and sarcopenia (binary; dependant variables) according to dietary patterns *z*-scores (independent variables) separately. Mild-moderate and severe malnutrition and probable sarcopenia, sarcopenia and severe sarcopenia were grouped to form binary variables herein termed ‘malnutrition’ and ‘(probable)-sarcopenia’, respectively. Analyses were adjusted for potential confounding factors identified prior to analysis using a directed acyclic graph (Figure S1). Confounders included age (continuous), sex (binary), smoking status (categorical), Townsend Deprivation Index (continuous), cancer type (categorical), physical activity level (categorical) and time since cancer diagnosis (years; continuous). Low energy intake was used as a proxy for ‘reduced food intake’ according to the GLIM definition of malnutrition. As such, models were not adjusted for energy when examining malnutrition. A sensitivity analysis was conducted where multilogistic regression models included energy intake (kJ; continuous) to determine the effects of energy intake on the association between dietary patterns and low muscle mass and (probable)-sarcopenia. BMI was identified in the directed acyclic graph (Figure S1) as a potential mediator. To prevent over-adjustment, models were not adjusted for BMI when BMI was already adjusted for as a component of the outcome (i.e. ALST/BMI). The creation of dietary patterns and the above regression models were re-run in the sub-sample of participants with a recent cancer diagnosis. Due to the limited number of cases of malnutrition, low muscle mass and/or sarcopenia in the sub-sample, regression models did not include ‘cancer type’ as a covariate. Tuckers’ coefficient of congruence estimated the similarity between dietary patterns derived from the full sample and sub-sample, based on food group factor loadings [35]. Coefficients of *r* ≥ 0.95 (equivalent) and *r* = 0.85–0.95 (similar) have been established previously [36]. Dietary patterns were generated using Statistical Analysis System On Demand (SAS Institute). All other statistical analyses were performed using Stata/MP 17 (Stata Corp) and food group factor loadings were graphically represented using Microsoft Excel. *P*-values < 0.05 were considered statistically significant.

### Exploratory analyses

In the event that any dietary pattern was associated with malnutrition, an investigation determined whether these relationships were driven by individual GLIM criteria. Logistic regression models investigated associations between dietary pattern *z*-scores (independent variables) and weight loss, low BMI, reduced energy intake and inflammation (binary; dependant variables) separately. Analyses were adjusted for potentially confounding factors identified prior to analysis using a directed acyclic graph (Figure S1).

## Results

Of the 502,493 UK Biobank participants, 500,078 participants were excluded based on the following: (i) they had no previous cancer diagnosis (*n* = 450,976; 89.7%); (ii) they had a cancer diagnosis after the baseline assessment or the Oxford WebQ dietary assessment not completed at the baseline assessment (*n* = 48,148; 9.6%); or (iii) they had missing data (outcomes, exposures, covariates) or implausible energy intakes (*n* = 954; 0.2%). A total of 2415 participants were included in the present analysis, from which 491 participants had a cancer diagnosis within 2 years of baseline assessment and were included in the sub-sample (Figure S2). Characteristics were similar between included participants and those with cancer who were excluded from the present analysis (*n* = 49,102) (Table S3).

### Participant characteristics

Table 2 presents the characteristics of all participants overall and by sex. Participants were aged (mean ± SD) 59.7 ± 7.1 years, with the majority being women (60.7%) and from Anglo-Saxon ethnic background (96.8%). Breast cancer (35.1%), genitourinary (21.7%) and gastrointestinal (11.8%) cancers were most common. Time since cancer diagnosis was (mean ± SD) 7.1 ± 6.3 years. Prevalence of malnutrition, low muscle mass and (probable)-sarcopenia were 17.7%, 5.0% and 6.5%, respectively.

**Table 2:** Baseline characteristics of UK Biobank participants with a previous cancer diagnosis and recent cancer diagnosis, overall and by sex

Characteristics	Previous diagnosis (*n* = 2415)			Recent diagnosis (*n* = 491)		
	Total	Male	Female	Total	Male	Female
*N* (%)	2415 (100.0)	950 (39.3)	1465 (60.7)	491 (100.0)	256 (52.1)	235 (47.7)
Age (years), mean ± SD	59.7 ± 7.1	61.3 ± 6.7	58.7 ± 7.1	59.4 ± 7.2	61.1 ± 6.5	57.6 ± 7.4
Cancer type, *n* (%)						
Bone and soft tissue	19 (0.8)	15 (1.6)	4 (0.3)	5 (1.0)	5 (2.0)	0 (0.0)
Breast	847 (35.1)	8 (0.8)	839 (57.3)	116 (23.6)	1 (0.4)	115 (48.9)
Central or peripheral nervous system	25 (1.0)	13 (1.4)	12 (0.8)	3 (0.6)	1 (0.4)	2 (0.9)
Endocrine and thyroid	43 (1.8)	10 (1.1)	33 (2.3)	6 (1.2)	2 (0.8)	4 (1.7)
Gastrointestinal	285 (11.8)	165 (17.4)	120 (8.2)	86 (17.5)	50 (19.5)	36 (15.3)
Genitourinary	523 (21.7)	497 (52.3)	26 (1.8)	143 (29.1)	138 (53.9)	5 (2.1)
Gynaecological	183 (7.6)	N/A	183 (12.5)	24 (4.9)	N/A	24 (10.2)
Haematological	170 (7.0)	99 (10.4)	71 (4.9)	36 (7.3)	23 (9.0)	13 (5.5)
Head and neck	63 (2.6)	39 (4.1)	24 (1.6)	14 (2.9)	8 (3.1)	6 (2.6)
Lung and other thoracic	23 (1.0)	14 (1.5)	9 (0.6)	8 (1.6)	4 (1.6)	4 (1.7)
Melanoma	214 (8.9)	82 (8.6)	132 (9.0)	47 (9.6)	23 (9.0)	24 (10.2)
Unknown primary	20 (0.8)	8 (0.8)	12 (0.8)	3 (0.6)	1 (0.4)	2 (0.9)
Years since cancer diagnosis, mean ± SD^1^	7.1 ± 6.3	5.9 ± 5.9	7.8 ± 6.5	1.0 ± 0.5	1.0 ± 0.5	1.1 ± 0.1
Ethnic background, *n* (%)						
Prefer not to answer	4 (0.2)	1 (0.1)	3 (0.2)	0 (0.0)	0 (0.0)	0 (0.0)
Anglo-Saxon (white, British, Irish)	2337 (96.8)	923 (97.2)	1414 (96.5)	473 (96.3)	247 (96.5)	226 (96.2)
Other	74 (3.1)	26 (2.7)	48 (3.3)	18 (3.7)	9 (3.5)	9 (3.8)
Townsend Deprivation Index, mean ± SD	−1.4 ± 2.8	−1.4 ± 2.8	−1.4 ± 2.8	−1.4 ± 2.7	−1.5 ± 2.7	−1.4 ± 2.8
Smoking status, *n* (%)						
Never	1291 (53.5)	443 (46.6)	848 (57.9)	261 (53.2)	120 (46.9)	141 (60.0)
Previous	958 (39.7)	440 (46.3)	518 (35.4)	196 (39.9)	118 (46.1)	78 (33.2)
Current	166 (6.9)	67 (7.1)	99 (6.8)	34 (6.9)	18 (7.0)	16 (6.8)
IPAQ physical activity group, *n* (%)^2^						
Low	425 (17.6)	167 (17.6)	258 (17.6)	90 (18.3)	39 (15.2)	51 (21.7)
Moderate	1043 (43.2)	388 (40.8)	655 (44.7)	204 (41.6)	102 (39.8)	102 (43.4)
High	947 (39.2)	395 (41.6)	552 (37.7)	197 (40.1)	115 (44.9)	82 (34.9)
BMI (kg/m^2^), mean ± SD^3^	27.1 ± 4.6	27.6 ± 3.9	26.8 ± 4.9	27.2 ± 4.7	27.5 ± 3.8	26.9 ± 5.5
Overweight, *n* (%)	1584 (65.6)	707 (74.4)	877 (59.9)	331 (67.4)	196 (76.6)	135 (57.5)
Obesity, *n* (%)	540 (22.4)	234 (24.6)	306 (20.9)	115 (23.4)	61 (23.8)	54 (23.0)
Energy intake (kJ/day), mean ± SD	8477 ± 2616	9457 ± 2850	7842 ± 2235	8734 ± 2754	9496 ± 2858	7904 ± 2378
Malnutrition, *n* (%)^4^						
Well nourished	1987 (82.3)	772 (81.3)	1215 (82.9)	399 (81.3)	206 (80.5)	193 (82.1)
Malnourished	428 (17.7)	178 (18.7)	250 (17.1)	92 (18.7)	50 (19.5)	42 (17.9)
Mild-moderate	357 (14.8)	141 (14.8)	216 (14.7)	77 (15.7)	42 (16.4)	35 (14.9)
Severe	71 (2.9)	37 (3.9)	34 (2.3)	15 (3.1)	8 (3.2)	7 (3.0)
Muscle mass, *n* (%)^5^						
Normal muscle mass	2295 (95.0)	873 (91.9)	1422 (97.1)	460 (93.7)	235 (91.8)	225 (95.7)
Low muscle mass	120 (5.0)	77 (8.1)	43 (2.9)	31 (6.3)	21 (8.2)	10 (4.3)
Sarcopenia, *n* (%)^6^						
Non-sarcopenic	2258 (93.5)	890 (93.7)	1368 (93.4)	450 (91.7)	238 (93.0)	212 (90.2)
(Probable)-sarcopenia	157 (6.5)	60 (6.3)	97 (6.6)	41 (8.4)	18 (7.0)	23 (9.8)
Probable sarcopenia	142 (5.9)	46 (4.8)	96 (6.6)	35 (7.1)	12 (4.7)	23 (9.8)
Sarcopenia	13 (0.5)	12 (1.3)	1 (0.1)	5 (1.0)	5 (2.0)	0 (0.0)
Severe sarcopenia	2 (0.1)	2 (0.2)	0 (0.0)	1 (0.2)	1 (0.4)	0 (0.0)

Data are reported as *n* (%) and mean ± SD. Abbreviations: *ALST*, appendicular lean soft tissue; *BMI*, body mass index; *IPAQ*, International Physical Activity Questionnaire

^1^Years since diagnosis: number of years from date of first cancer diagnosis to date of baseline assessment visit

^2^IPAQ physical activity group: ‘low physical activity group’, ≤ 918 MET minute/week; ‘high physical activity group’, > 3706–19,278 MET minute/week

^3^Overweight and obesity (BMI): classified as body mass index ≥ 25 kg/m^2^ and body mass index ≥ 30 kg/m^2^, respectively

^4^Malnutrition: amended definition according to the Global Leadership Initiative on Malnutrition criteria. Severe malnutrition defined as malnourishment with BMI < 18.5 kg/m^2^ (< 70 years) and < 20 kg/m^2^ (≥ 70 years) or ALST/BMI < 0.84 (male) and < 0.55 (female)

^5^Low muscle mass defined as ALST/BMI < 0.84 (males) and < 0.55 (females)

^6^Sarcopenia: amended definition according to European Working Group on Sarcopenia in Older People 2019 definitions. Probable sarcopenia, handgrip strength < 27 kg (male) and < 16 kg (female); sarcopenia, low handgrip strength plus ALST/BMI < 0.84 (male) and < 0.55 (female); severe sarcopenia, low handgrip strength plus low muscle mass (ALST/BMI) plus low physical performance (slow walking speed)

The sub-sample of participants with a recent cancer diagnosis had a higher proportion of men (52.1%) than in the full sample. Genitourinary (29.1%), breast (23.6%) and gastrointestinal (17.5%) cancers were the most common cancer types, with time since diagnosis (mean ± SD) being 1.0 ± 0.5 years. Prevalence of malnutrition (18.7%), low muscle mass (6.3%) and (probable)-sarcopenia (8.4%) was higher compared to the full sample.

### Dietary pattern characteristics

**Fig. 1:**
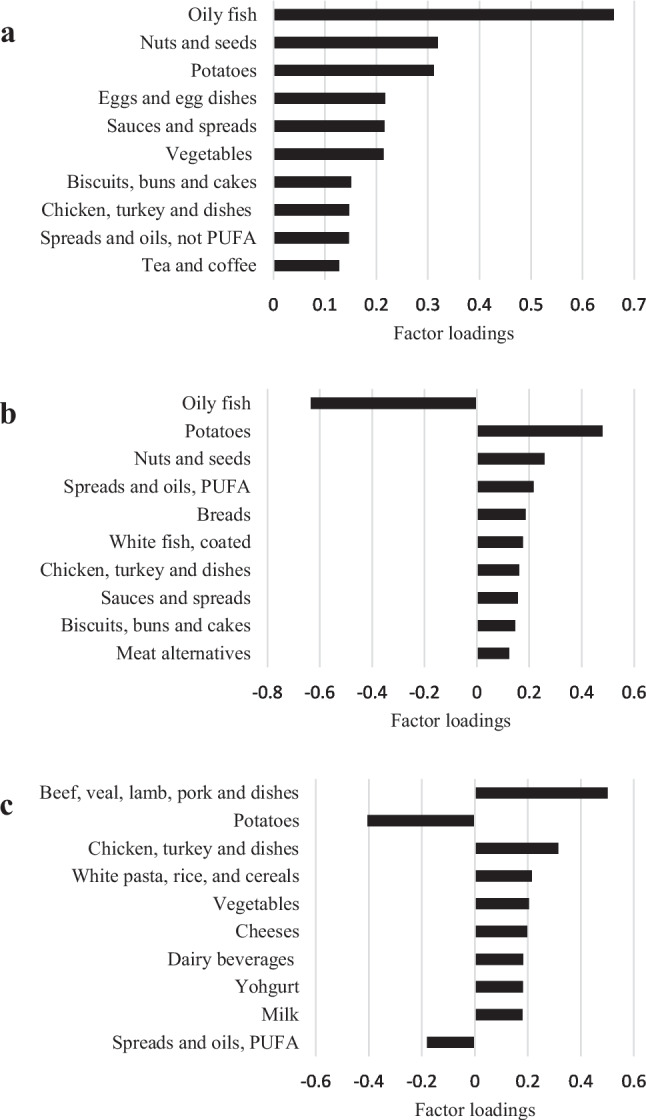
Highest (absolute) factor loading food groups for ‘high oily fish and nuts’ (**a**), ‘low oily fish’ (**b**) and ‘meat and dairy’ (**c**) dietary patterns in UK Biobank participants with a previous cancer diagnosis (*n* = 2415)

Three dietary patterns were derived from the full sample (‘high oily fish and nuts’, ‘low oily fish’, ‘meat and dairy’) and sub-sample of participants with a recent cancer diagnosis (‘high oily fish and nuts—sub’, ‘low oily fish—sub’, ‘meat and dairy—sub’). All dietary patterns explained more than 10% of the variation in the response variable intake and were considered for analysis (Table S4). Table S5 presents a complete list of food group factor loadings which were equivalent between dietary patterns derived from the full sample and sub-sample (‘high oily fish and nuts’: *r*_c_ = 0.97; ‘low oily fish’: *r*_c_ = 0.96; ‘meat and dairy’: *r*_c_ = 0.95). The 10 food groups with the strongest factor loadings (positive or negative) are presented in Fig. 1 and Table 3.

**Table 3:** Response variable, energy, nutrient and key food group intakes across tertiles of dietary pattern scores in UK Biobank participants with a previous cancer diagnosis (*n* = 2415)

	Dietary pattern scores					
	Tertile 1 (*n* = 805)		Tertile 2 (*n* = 805)		Tertile 3 (*n* = 805)	
	Mean ± SD	Median (IQR)	Mean ± SD	Median (IQR)	Mean ± SD	Median (IQR)
*‘High oily fish and nuts’ pattern*						
Response variables						
Protein (g/kg/day)	0.79 ± 0.28	0.77 (0.59, 0.96)	1.09 ± 0.29	1.06 (0.88, 1.26)	1.30 ± 0.35	1.25 (1.04, 1.52)
PUFA (g/day)	8.06 ± 4.66	7.05 (4.65, 10.46)	13.13 ± 5.76	12.07 (8.71, 17.04)	20.08 ± 8.89	18.99 (13.31, 25.20)
Vitamin D (μg/day)	1.14 ± 0.10	0.96 (0.42, 1.6)	2.01 ± 1.71	1.63 (0.94, 2.62)	5.57 ± 4.52	3.89 (2.05, 9.20)
Foods (g/day)						
Oily fish (+)	0.1 ± 2.7	0 (0, 0)	4.4 ± 19.5	0 (0, 0)	54.3 ± 65.9	0 (0, 114.0)
Nuts and seeds (+)	1.5 ± 6.5	0 (0, 0)	5.0 ± 13.0	0 (0, 0)	12.8 ± 26.1	0 (0, 16.0)
Potatoes (+)	3.6 ± 19.7	0 (0, 0)	17.2 ± 43.5	0 (0, 0)	40.0 ± 76.2	0 (0, 68.8)
Eggs and dishes (+)	9.3 ± 28.3	0 (0, 0)	19.8 ± 45.0	0 (0, 0)	36.2 ± 67.2	0 (0, 53)
Sauces and spreads (+)	11.7 ± 15.6	0 (0, 32.5)	18.4 ± 16.1	32.5 (32.5, 32.5)	19.8 ± 15.9	32.5 (32.5, 32.5)
Vegetables (+)	207.1 ± 195.8	176.3 (0, 327.5)	270.0 ± 228.0	241.3 (85.0, 410.0)	312.1 ± 258.1	272.5 (120.0, 451.3)
Biscuits, buns and cakes (+)	24.2 ± 46.1	0 (0, 30.0)	41.0 ± 62.1	0 (0, 61.3)	52.9 ± 81.0	2.5 (0, 110.0)
Chicken, turkey and dishes (+)	18.8 ± 47.3	0 (0, 0)	34.5 ± 63.5	0 (0, 0)	39.9 ± 75.6	0 (0, 0)
Spreads and oils, not PUFA (+)	2.4 ± 7.1	0 (0, 0)	4.7 ± 11.0	0 (0, 0)	5.3 ± 12.9	0 (0, 0)
Tea and coffee (+)	905 ± 454.7	900 (675.0, 1125.0)	1001.2 ± 434.4	1125 (675.0, 1350.0)	1040 ± 447.0	1125 (675.0, 1350.0)
Nutrients						
Total energy (kJ/day)	6727 ± 1990	6627 (5413, 7848)	8604 ± 2055	8437 (7200, 9748)	10,101 ± 2579	9893 (8384, 11,714)
Protein (% energy)	15.1 ± 4.5	14.5 (11.9, 17.6)	16.6 ± 4.0	16.3 (13.8, 18.8)	17.2 ± 4.1	16.7 (14.5, 19.1)
Total fat (% energy)	29.4 ± 8.7	29.7 (23.4, 34.8)	32.5 ± 7.5	32.5 (27.0, 37.4)	35.5 ± 7.6	35.4 (30.3, 40.3)
Saturated fat (% energy)	12.3 ± 4.6	12.3 (9.1, 15.2)	12.6 ± 4.0	12.5 (9.7, 15.3)	12.3 ± 3.8	12.1 (9.7, 14.8)
Carbohydrate (% energy)	53.3 ± 10.5	54.2 (46.7, 60.3)	50.2 ± 8.7	50.3 (44.7, 56.3)	46.4 ± 9.0	47.3 (41.0, 52.9)
Total sugars (% energy)	27.0 ± 9.3	26.5 (20.6, 32.5)	24.1 ± 7.8	23.9 (18.8, 29.3)	22.7 ± 7.3	22.5 (17.6, 27.2)
Dietary fibre (g/day)	13.5 ± 6.1	13.1 (8.9, 17.5)	17.0 ± 6.8	16.3 (12.4, 21.0)	19.7 ± 8.2	18.8 (14.2, 24.2)
*‘Low oily fish’ pattern*						
Response variables						
Protein (g/kg/day)	0.97 ± 0.39	0.95 (0.70, 1.20)	1.02 ± 0.33	0.99 (0.79, 1.21)	1.18 ± 0.36	1.15 (0.92, 1.40)
PUFA (g/day)	10.61 ± 6.84	8.83 (5.46, 14.21)	10.57 ± 5.47	9.41 (6.83, 12.99)	20.08 ± 8.37	18.73 (14.11, 24.45)
Vitamin D (μg/day)	5.08 ± 4.77	3.39 (1.28, 9.08)	1.78 ± 1.66	1.38 (0.73, 2.35)	1.86 ± 1.67	1.49 (0.83, 2.41)
Foods (g/day)						
Oily fish (−)	52.7 ± 65.8	0 (0, 114.0)	3.4 ± 17.9	0 (0, 0)	2.7 ± 16.3	0 (0, 0)
Potatoes (+)	3.0 ± 18.6	0 (0, 0)	3.2 ± 17.3	0 (0, 0)	54.6 ± 79.7	0 (0, 137.5)
Nuts and seeds (+)	3.2 ± 11.6	0 (0, 0)	3.1 ± 10.9	0 (0, 0)	13.1 ± 25.3	0 (0, 20.0)
Spreads and oils, PUFA (+)	2.2 ± 7.3	0 (0, 0)	3.5 ± 9.1	0 (0, 0)	9.0 ± 15.7	0 (0, 20.0)
Breads (+)	68.9 ± 69.1	72.0 (0.0, 108.0)	92.8 ± 70.7	72.0 (56.7, 120.0)	116.3 ± 106.2	108.0 (60.0, 144.0)
White fish, coated (+)	1.5 ± 14.1	0 (0, 0)	4.6 ± 27.3	0 (0, 0)	16.8 ± 59.6	0 (0, 0)
Chicken, turkey and dishes (+)	16.4 ± 49.7	0 (0, 0)	30.9 ± 59.6	0 (0, 0)	46.0 ± 76.0	0 (0, 152.3)
Sauces and spreads (+)	11.9 ± 15.7	0 (0, 32.5)	17.4 ± 16.2	32.5 (0, 32.5)	20.5 ± 15.7	32.5 (0, 32.5)
Biscuits, buns and cakes (+)	26.8 ± 47.8	0 (0, 30.0)	37.5 ± 61.1	0 (0, 55.0)	53.9 ± 81.0	7.5 (0, 110.0)
Meat alternatives (+)	0.6 ± 6.4	0 (0, 0)	2.2 ± 13.6	0 (0, 0)	6.2 ± 28.4	0 (0, 0)
Nutrients						
Total energy (kJ/day)	7452 ± 2466	7196 (5722, 9026)	8038 ± 2178	7744 (6524, 9298)	9942 ± 2517	9610 (8157, 11,460)
Protein (% energy)	16.8 ± 5.0	16.3 (13.3, 19.7)	16.4 ± 4.2	15.9 (13.5, 18.9)	15.7 ± 3.6	15.5 (13.2, 17.6)
Total fat (% energy)	31.5 ± 9.5	30.9 (25.0, 38.1)	31.5 ± 7.9	31.0 (26.3, 36.3)	34.4 ± 7.0	34.8 (29.8, 39.0)
Saturated fat (% energy)	12.3 ± 4.7	11.9 (9.0, 15.2)	12.8 ± 4.1	12.9 (9.9, 15.5)	12.1 ± 3.5	12.0 (9.7, 14.5)
Carbohydrate (% energy)	49.2 ± 11.5	49.3 (41.4, 56.8)	51.3 ± 9.2	51.6 (45.3, 57.8)	49.5 ± 8.4	49.7 (44.1, 55.3)
Total sugars (% energy)	26.0 ± 9.5	25.1 (19.4, 31.1)	25.1 ± 8.0	24.7 (19.6, 30.4)	22.8 ± 7.2	22.7 (17.6, 27.5)
Dietary fibre (g/day)	14.0 ± 6.9	13.2 (8.9, 18.3)	16.0 ± 6.4	15.5 (11.6, 19.7)	20.3 ± 7.8	19.0 (14.6, 24.6)
*‘Meat and dairy’ pattern*						
Response variables						
Protein (g/kg/day)	0.87 ± 0.34	0.86 (0.63, 1.07)	1.02 ± 0.32	0.99 (0.80, 1.22)	1.27 ± 0.35	1.22 (1.02, 1.46)
PUFA (g/day)	16.94 ± 9.77	16.12 (9.11, 22.77)	12.68 ± 7.36	11.08 (7.33, 16.8)	11.65 ± 6.44	10.43 (6.87, 15.26)
Vitamin D (μg/day)	3.51 ± 4.12	1.88 (0.84, 4.46)	2.75 ± 3.34	1.55 (0.78, 3.24)	2.45 ± 2.59	1.68 (0.98, 2.83)
Foods (g/day)						
Beef, veal, lamb and dishes (+)	13.6 ± 45.0	0 (0, 0)	37.5 ± 68.2	0 (0, 0)	104.7 ± 106.3	163.3 (0, 167.5)
Potatoes (−)	44.5 ± 76.1	0 (0, 137.5)	11.4 ± 37.4	0 (0, 0)	5.0 ± 26.0	0 (0, 0)
Chicken, turkey and dishes (+)	8.7 3 ± 5.4	0 (0, 0)	28.5 ± 57.4	0 (0, 0)	56.1 ± 81.0	0 (0, 152.3)
White pasta, rice and cereals (+)	21.8 ± 62.6	0 (0, 0)	41.8 ± 84.9	0 (0, 0)	58.6 ± 99.3	0 (0, 150.0)
Vegetables (+)	206.2 ± 206.0	175.0 (0, 326.3)	270.9 ± 233.4	237.5 (90.0, 405.7)	312.3 ± 244.7	283.8 (132.5, 442.5)
Cheeses (+)	9.9 ± 15.3	0 (0, 15)	14.3 ± 19.6	0 (0, 30)	18.5 ± 24.5	15 (0, 30)
Dairy beverages (+)	34.2 ± 97.0	0 (0, 0)	56.4 ± 127.6	0 (0, 0)	87.3 ± 178.9	0 (0, 225.0)
Yoghurt (+)	48.2 ± 98.5	0 (0, 0)	80.5 ± 121.8	0 (0, 125.0)	94.6 ± 136.8	0 (0, 250.0)
Milk (+)	141.3 ± 100.0	137.5 (82.5, 210.0)	167.2 ± 134.6	155.0 (100.0, 220.0)	190.0 ± 165.8	182.5 (110.0, 237.5)
Spreads and oils, PUFA (−)	7.3 ± 14.7	0 (0, 0)	4.1 ± 10.0	0 (0, 0)	3.3 ± 9.0	0 (0, 0)
Nutrients						
Total energy (kJ/day)	8233 ± 2720	8009 (6365, 9832)	8257 ± 2546	7846 (6391, 9816)	8943 ± 2519	8743 (7157, 10,279)
Protein (% energy)	13.6 ± 3.3	13.3 (11.4, 15.5)	16.1 ± 3.5	15.7 (13.6, 18.0)	19.1 ± 4.1	18.3 (16.3, 21.3)
Total fat (% energy)	34.6 ± 8.6	34.7 (29.2, 40.0)	32.1 ± 8.4	32.3 (26.2, 38.0)	30.8 ± 7.5	30.5 (25.9, 35.4)
Saturated fat (% energy)	12.2 ± 4.2	12.2 (9.4, 14.9)	12.5 ± 4.3	12.2 (9.4, 15.4)	12.5 ± 3.9	12.4 (9.8, 15.1)
Carbohydrate (% energy)	50.2 ± 10.1	50.1 (43.8, 57.0)	50.4 ± 10.1	50.7 (43.6, 57.3)	49.4 ± 9.3	49.9 (43.9, 55.6)
Total sugars (% energy)	24.0 ± 8.7	23.3 (18.0, 29.1)	24.7 ± 8.2	24.1 (19.3, 29.6)	25.1 ± 8.1	24.7 (19.5, 30.3)
Dietary fibre (g/day)	15.9 ± 7.1	15.3 (11.0, 19.9)	16.6 ± 7.3	15.9 (11.7, 20.6)	17.8 ± 8.0	17.1 (12.3, 22.1)

Abbreviations: *PUFA*, polyunsaturated fatty acids. Represented foods have the highest factor loading (positive [+] or negative [−]) for the respective dietary pattern

Non-consumers (*n*, %): oily fish (1996, 82.7); nuts and seeds (2037, 84.3); potatoes (2045, 84.7); sauces and spreads (1181, 48.9); eggs and dishes (1883, 78.0); vegetables (496, 20.5); biscuits, buns and cakes (1331, 55.1); chicken, turkey and dishes (1902, 78.8); spreads and oils, not PUFA (2008, 83.2); tea and coffee (102, 4.2); spreads and oils, PUFA (1957, 81.0); breads (439, 18.2); white fish, coated (2308, 95.6); meat alternatives (2339, 96.9) beef, veal, lamb and pork (1692, 70.1); white pasta, rice and cereals (1916, 79.3); cheeses (1326, 54.9); dairy beverages (1918, 79.4); yoghurt (1606, 66.5); milk (275; 11.4)

### ‘High oily fish and nuts’ dietary patterns

The ‘high oily fish and nuts’ pattern was moderately positively correlated with vitamin D (*r* = 0.61), PUFA (*r* = 0.58) and protein (*r* = 0.54) and was characterised by high intakes of oily fish (i.e. salmon, herring, mackerel sardines and tuna), nuts and seeds and potatoes (i.e. fried, chips, wedges and roasted potatoes) (Fig. 1a). The highest tertile of ‘high oily fish and nuts’ pattern consumed the highest mean total energy, percentage energy (%E) from protein and total fat and lowest %E from carbohydrates and total sugars compared to the middle and lowest tertiles. Table 3 presents response variables, key food groups, energy and nutrient intakes across tertiles of dietary pattern scores. In the sub-sample of participants with a recent cancer diagnosis, the ‘high oily fish and nuts—sub’ pattern was moderately positively associated with vitamin D (*r* = 0.64), PUFA (*r* = 0.59) and protein (*r* = 0.49). Notably, intake of key food groups (Figure S3), response variables, energy and nutrients (Table S6) were similar between the dietary patterns derived from the full sample and sub-sample, respectively.

### ‘Low oily fish’ dietary patterns

The ‘low oily fish’ pattern was moderately positively correlated with PUFA (*r* = 0.61), weakly positively correlated with protein (*r* = 0.21) and strongly negatively correlated with vitamin D (*r* = −0.76). The ‘low oily fish’ pattern was best characterised by low intake of oily fish but high intake of potatoes, nuts and seeds and PUFA fats and oils (i.e. margarine and sunflower oil) (Fig. 1b). The highest tertile consumed the highest mean total energy, %E from total fat, and lowest %E from total sugars compared to the lowest and middle tertiles. The middle tertile consumed the highest mean %E from carbohydrates (Table 3). In the sub-sample of participants with a recent cancer diagnosis, the ‘low oily fish—sub’ pattern was moderately positively correlated with PUFA (*r* = 0.55), weakly positively correlated with protein (*r* = 0.33) and strongly negatively correlated with vitamin D (*r* = −0.77).

### ‘Meat and dairy’ dietary patterns

The ‘meat and dairy’ pattern was strongly positively correlated with protein (*r* = 0.82), moderately negatively correlated with PUFA (*r* = −0.54) and weakly negatively correlated with vitamin D (*r* = −0.21). The ‘meat and dairy’ pattern was characterised by high intake of meat, poultry, white pasta and rice, vegetables and dairy foods (cheese, dairy beverages, yoghurt and milk) and low intake of potatoes (Fig. 1c). The highest tertile consumed the highest mean %E from protein and total sugars and lowest %E from total fat and carbohydrates compared to the middle and lowest tertiles (Table 3). In the sub-sample of participants with a recent cancer diagnosis, the ‘meat and dairy—sub’ pattern was strongly positively correlated with protein (*r* = 0.80), moderately negatively correlated with PUFA (*r* = −0.59) and weakly negatively correlated with vitamin D (*r* = −0.07).

### Dietary patterns and associations with malnutrition, low muscle mass or sarcopenia

As shown in Table 4, higher dietary pattern *z*-scores for the ‘high oily fish and nuts’ (OR = 0.57 [95% CI 0.50, 0.65]) and ‘low oily fish’ (OR = 0.81 [95% CI: 0.73, 0.90]) patterns, but not the ‘meat and dairy’ pattern, were associated with significantly lower odds of malnutrition. In the sub-sample of participants with a recent cancer diagnosis, higher dietary pattern *z*-scores for the ‘high oily fish and nuts—sub’ (OR = 0.53 [95% CI 0.39, 0.72]) and ‘low oily fish—sub’ (OR = 0.78 [95% CI: 0.63, 0.98]) patterns, but not ‘meat and dairy—sub’ pattern, were associated with significantly lower odds of malnutrition. No dietary patterns were significantly associated with low muscle mass or sarcopenia. The sensitivity analysis demonstrated that adjusting for energy intake (kJ) did not substantially alter the associations between dietary patterns and low muscle massand (probable)-sarcopenia (Table S7).

**Table 4:** Odds ratios (OR) and 95% confidence intervals (CI) for associations between dietary patterns and prevalent malnutrition, low muscle mass and sarcopenia at the baseline assessment for UK Biobank participants with a previous cancer diagnosis and recent cancer diagnosis

Dietary patterns	Malnutrition^1^		Low muscle mass^2^		Sarcopenia^3^	
	OR (95% CI)	*P*-value	OR (95% CI)	*P*-value	OR (95% CI)	*P*-value
Previous diagnosis (*n* = 2415)						
High oily fish and nuts	0.57 (0.50, 0.65)	< 0.001	0.87 (0.72, 1.06)	0.17	1.10 (0.95, 1.29)	0.21
Low oily fish	0.81 (0.73, 0.90)	< 0.001	0.93 (0.78, 1.12)	0.44	1.06 (0.90, 1.24)	0.52
Meat and dairy	0.94 (0.85, 1.05)	0.28	0.95 (0.80, 1.14)	0.61	0.89 (0.75, 1.05)	0.17
Recent diagnosis (*n* = 491)						
High oily fish and nuts—sub	0.53 (0.39, 0.72)	< 0.001	0.71 (0.46, 1.11)	0.13	1.02 (0.73, 1.41)	0.91
Low oily fish—sub	0.78 (0.63, 0.98)	0.03	1.07 (0.74, 1.53)	0.73	0.82 (0.60, 1.12)	0.21
Meat and dairy—sub	0.89 (0.71, 1.13)	0.34	1.02 (0.71, 1.46)	0.93	0.89 (0.64, 1.25)	0.50

Abbreviations: *CI*, confidence interval; *OR*, odds ratio

^1^Malnutrition: amended definition according to the Global Leadership Initiative on Malnutrition criteria. Low ALST/BMI for reduced muscle mass criteria. Mild-moderate and severe malnutrition diagnoses were combined

^2^Low muscle mass: ALST/BMI

^3^Sarcopenia: amended definition according to the European Working Group on Sarcopenia in Older People 2019 definitions. Low ALST/BMI for low muscle mass criteria. Probable, sarcopenia and severe sarcopenia diagnoses combined

Model adjusted for age, sex, smoking status, Townsend Deprivation Status, cancer type, physical activity level and time since diagnosis (full sample), or age, sex, smoking status, Townsend Deprivation Status, physical activity level and time since diagnosis (sub-sample)

### Exploratory analysis

As shown in Table S8, higher dietary pattern *z*-scores for ‘high oily fish and nuts’, ‘low oily fish’ and ‘meat and dairy’ patterns were associated with significantly lower odds of low energy intake (‘high oily fish and nuts’: OR = 0.28 [95% CI: 0.24, 0.33]; ‘low oily fish’: OR = 0.60 [95% CI: 0.54, 0.67]; ‘meat and dairy’: OR = 0.81 [95% CI: 0.74, 0.89]). No dietary patterns were significantly associated with inflammation, body weight or BMI. In the sub-sample, higher dietary pattern *z*-scores for all three dietary patterns were associated with significantly lower odds of low energy intake (‘high oily fish and nuts—sub’: OR = 0.27 [95% CI: 0.19, 0.38]; ‘low oily fish—sub’: OR = 0.59 [95% CI: 0.47, 0.73]; ‘meat and dairy—sub’: OR = 0.79 [95% CI: 0.64, 0.97]). No other statistically significant relationships were observed.

## Discussion

This cross-sectional cohort study in cancer survivors identified three dietary patterns associated with protein, PUFA and vitamin D intakes. Energy-dense dietary patterns characterised by high vitamin D, PUFA and protein intakes (from high intake of oily fish and nuts and seeds) and high PUFA and protein but low vitamin D intakes (from low oily fish and high fried potatoes intake) were associated with a 19 to 43% lower odds of malnutrition. However, no dietary patterns were associated with low muscle mass or (probable)-sarcopenia. This study is the first to demonstrate that RRR-derived dietary patterns, based on cancer-specific nutritional recommendations, are related to lower odds of malnutrition in cancer survivors. These findings highlight the potential to incorporate dietary patterns as a novel approach to managing malnutrition within the clinical setting.

The present study identified two dietary patterns (‘high oily fish and nuts’ and ‘low oily fish’ patterns’) associated with significantly lower odds of malnutrition. To the authors’ knowledge, this is the first study to investigate data-driven dietary patterns in relation to nutritional status in cancer. Our findings support the body of evidence for the vital role of nutrition in improving malnutrition. In a recent large-scale randomised controlled trial, improved survival was observed in hospitalised patients (20% of the intervention group had an admission diagnosis of cancer) receiving individualised nutritional support to meet protein and caloric goals [37]. Individualised nutritional support was provided in a stepwise escalation from food-based strategies through to enteral and parental nutrition [37]. This individualised escalation approach to nutrition was also shown to reduce mortality risk and improve function and QOL outcomes in patients with cancer, demonstrating the benefits of optimising nutritional status on patient outcomes [38]. In clinical practise, a high-energy, high-protein diet is commonly recommended as a first-line approach to achieve nutrient targets and treat cancer-related malnutrition during treatment and into survivorship [39]. However, the combination of foods and beverages which optimally support nutritional status in cancer has remained uncertain. As a result, we have provided novel evidence of dietary patterns characterised by energy-rich foods and beverages which support the initial food-first step to manage malnutrition. Further research should determine whether replacing highly palatable, energy-dense foods with more nutritious high-energy options will be advantageous in managing malnutrition and translate into health benefits such as preventing or reducing loss of muscle mass and the risk of sarcopenia.

There are a number of potential mechanistic explanations for why dietary patterns characterised by protein, PUFA and vitamin D may be associated with lower odds of malnutrition. Evidence from supplementation studies suggests prolonged omega-3 fatty acid intake may improve food intake, appetite, body weight and QOL in cancer [40]. The mechanistic effects of vitamin D, such as calcium and phosphate regulation and regulation of gene-transcription involved in muscle protein synthesis, differentiation and proliferation, may also play a pivotal role in malnutrition prevention [7]. The present study identified two dietary patterns, both associated with lower odds of malnutrition, characterised by high intake of PUFA but characterised by opposing vitamin D and oily fish intakes. As a result, the present study has highlighted the importance of food sources on the mechanistic effects of individual nutrients. Specifically, the combined role of nutrients and non-nutritive components within a food matrix support the role for a food-based approach to understanding malnutrition prevention and treatment.

Our findings for no effect on muscle mass with adherence to a dietary pattern characterised by positive factor loadings for foods such as oily fish, nuts and seeds and vegetables are consistent with previous research of a priori dietary patterns, the Mediterranean Diet and Healthy Eating Index 2010 [41, 42]. In consensus, the Mediterranean Diet and Healthy Eating Index 2010 have previously been shown not to be associated with or detrimental to muscle mass in people with cancer [41, 42]. Whilst our dietary patterns were associated with adequate protein intake (~1.3 g/kg/day), according to evidence-based recommendations (1.0–1.5 g/kg/day) to support muscle health [4], these recommendations are subject to scrutiny, where protein intakes of up to 2 g/kg/day have been proposed to combat these effects in adults with cancer [43]. Thus, it is plausible that our dietary patterns contained inadequate protein-rich food sources (or doses of protein) to prevent losses of muscle mass or strength in patients experiencing the catabolic effects of cancer. Moreover, ‘healthful’ dietary patterns developed for the general population and characterised by food sources such as lean protein, fruits and vegetables and wholegrains may be inadequate to meet the nutritional requirements of cancer survivors who remain at risk of malnutrition. As such, an optimal dietary pattern for the protection of muscle mass and strength in cancer remains unclear.

### Strength and limitations

This study provided novel information on the combination of foods and beverages to best prevent malnutrition in cancer survivors. To the best of our knowledge, this is the first study to derive dietary patterns based on nutrients of interest from current nutritional guidelines and examine their associations with malnutrition, low muscle mass and sarcopenia in a large cohort of cancer survivors. However, reverse causality cannot be discounted due to the cross-sectional study design. The limited sample size (0.5% of the participants in the UK Biobank) may also limit the generalisability of our findings, especially in the analysis of those with a recent cancer diagnosis. The limited sub-sample precluded the use of the same confounders accounted for in the analysis of the full sample. One dietary recall was used to estimate dietary intake at the baseline assessment. Single 24-h recalls have previously been considered valid compared to observed dietary intake, especially in groups [44]. Notably, when comparing reported energy intake from a single 24-h recall with that from multiple days, accuracy does not significantly improve with increased number of days [45]. Whilst the 24-h recall is regarded as a valid approach, the use of one dietary recall to estimate dietary intake has limitations in terms of assessing ‘usual’ dietary intake and may introduce random errors. It is also noted that the use of a single 24-h dietary recall may incorrectly identify ‘non-consumers’ of certain episodically consumed foods (i.e. oily fish). Therefore, it is recommended that future studies consider the use of multiple dietary assessments if this approach is clinically relevant. The dietary patterns are cohort-specific, limiting their generalisability to other population groups. Generation of food groups was ultimately selected by researchers and may be biased by subjectivity, where a different concoction of food groups and/or nutrient response variables may result in distinctly different patterns. Overall energy intake (kJ) is a potential driving factor for the associations between the high-energy dietary patterns (i.e. high oily fish and nuts) and malnutrition. The use of energy intake (kJ/kg/day) as a proxy for ‘reduced food intake’ in the diagnosis of malnutrition precluded the use of energy intake (kJ) as a confounder in our analysis. However, future studies should use an alternative method of estimating ‘reduced food intake’ to subsequently allow for the assessment of energy as a potential confounder in analysis. Finally, low muscle mass (ALST) was estimated from bioimpedance analysis which has known limitations and then derived using a prediction equation developed in a non-cancer population, which has potential to introduce error. The adapted definitions for malnutrition, low muscle mass and sarcopenia reflect the availability of data in the UK Biobank study but may have implications for the true prevalence of these outcomes.

### Implications for practise and future directions

The present study builds upon the current body of nutrient-centric literature and nutritional guidelines by uncovering a beneficial combination of foods and beverages most protective of malnutrition in cancer survivors. These findings demonstrate the potential to incorporate dietary patterns as a novel approach to nutritional interventions in the clinical setting. Future research should establish whether swapping energy-dense foods such as fried potatoes and cakes and biscuits for more nutritious but similarly calorific food sources will result in an optimal dietary pattern to prevent malnutrition in cancer. Prospective research is required to confirm these findings in various cancer types. Whether the observed positive effects on nutritional status equate to improved longer term outcomes such as survival should be examined in this cohort.

### Conclusion

Energy-rich dietary patterns characterised by high intakes of oily fish, nuts and seeds and potatoes or low oily fish but high intake of potatoes and nuts and seeds were associated with lower odds of malnutrition in people with a previous cancer diagnosis, including those diagnosed with cancer in the previous 2 years. However, no dietary patterns were found to be associated with a reduced risk of low muscle mass or sarcopenia. This study complements current evidence-based nutritional guidelines and provides guidance on a food-first approach to managing malnutrition during treatment and into survivorship. As a novel approach to treating cancer-related malnutrition, further research is required to determine the optimal dietary pattern/s for people with cancer and whether these dietary patterns are related to important clinical outcomes such as survival.

## Supplementary information

ESM 1 (DOCX 204 KB)
