# Supplementary material for: Dietary patterns, malnutrition, muscle loss and sarcopenia in cancer survivors: findings from the UK Biobank
Source: J Cancer Surviv. 2023 Jul 20;18(6):1889–902. doi: 10.1007/s11764-023-01428-8 (PMC11502595; doi:10.1007/s11764-023-01428-8)
Supplement: Supplementary file 1 — ESM 1 (DOCX 204 KB) [file 11764_2023_1428_MOESM1_ESM.docx]

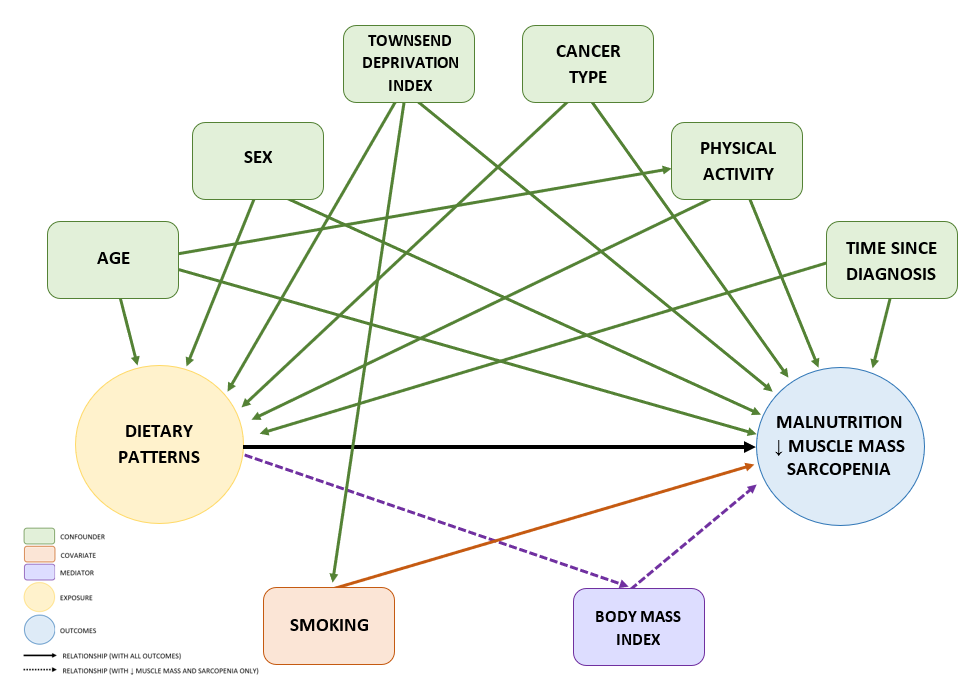


**Figure S1** Directed Acyclic Graph of proposed relationships between dietary patterns (exposure) and malnutrition, low muscle mass and (probable)sarcopenia (outcomes). This figure includes the casual pathways as well as potential confounders (age, sex, Townsend Deprivation Index, cancer types, physical activity level and time since diagnosis), covariates (smoking status), and mediators (body mass index). Models were not adjusted for BMI where muscle mass was already adjusted for BMI (i.e., ALST/BMI).

**UK Biobank participants**

*N* = 502,493

**Participants with cancer**

(excl. non-melanoma skin cancer and benign tumours)

*N* = 51,517

**Ineligible participants**

*N* = 48,148

Cancer diagnosed after initial assessment visit (*N* = 28,181)

Did not complete Oxford WebQ at baseline (*N* = 19,967)

**Missing or invalid data**

*N* = 954

Missing data for outcomes, exposures, or covariates (*N* = 890)

Invalid energy intake (*N* = 64)

**Population for full sample analysis**

*N* = 2,415

**Eligible participants**

*N* = 3,369

**Population for sub sample analysis**

*N* = 491

**Cancer diagnosis >2 years prior to initial assessment visit**

*N* = 1,924

**No cancer diagnosis**

*N* = 450,976

**Figure S2** Flow diagram of eligible participants from the UK Biobank cohort

**a**

**b**

**c**

**Figure S3** Highest (absolute) factor loadings for the ‘high oily fish and nuts -sub’ (a), ‘low oily fish - sub’ (b) and ‘meat and dairy - sub’ (c) dietary patterns in UK Biobank participants with a recent cancer diagnosis (n = 491)

| **Table S1** Cancer types according to ICD-10 code | | |
| --- | --- | --- |
| **Cancer type** | **Included cancers** | **ICD-10 codes** |
| Bone and soft tissue | Bone, articular cartilage of limbs and other unspecified sites, Kaposi sarcoma, other connective and soft tissue | C40, C41, C46, C49  (C40.0, C40.1, C40.2, C40.3, C40.8, C40.9)  (C41.0, C41.1, C41.2, C41.3, C41.4, C41.8, C41.9)  (C46.0 – C46.9, excl C46.4, C46.5, C46.6)  (C49.0 – C49.9, excl C49.7) |
| Breast | Breast | C50 (C50.0 – C50.9 excl C50.7) |
| Central and peripheral nervous system | Eye, brain, other parts of the CNS, peripheral nerves, autonomic nervous system | C69, C70, C71, C72, C47  (C69.0 – C69.9 excl C69.7) (C70.0, C70.1, C70.9)  (C71.0 – C71.9)  (C72.0 – C72.9, excl C72.6, C72.7)  (C47.0 – C47.9 excl C47.7) |
| Endocrine and thyroid | Thyroid, adrenal gland, other endocrine glands and related structures | C73, C74, C75  (C74.0, C74.1, C74.9)  (C75.0 – C75.9 excl C75.6, C75.7) |
| Gastrointestinal | Oesophagus, stomach, small intestine, colon, rectoid-sigmoid junction, rectum, anus, anal canal, liver, intrahepatic bile ducts, gallbladder, unspecified parts of biliary tract, pancreas, other and ill-defined digestive organs, retroperitoneum and peritoneum, | C15, C16, C17, C18, C19, C20, C21, C22, C23, C24, C25, C26, C48  (C15.0 – C15.9, excl C15.6, C15.7)  (C16.0 – C16.9, excl C16.7)  (C17.0, C17.1, C17.2, C17.3, C17.8, C17.9)  (C18.0 – C18.9)  (C21.0, C21.1, C21.2 C21.8)  (C22.0, C22.1, C22.2, C22.3, C22.4, C22.7, C22.9)  (C24.0, C24.1, C24.8, C24.9)  (C25.0 – C25.9 excl C25.5, C25.6)  (C26.0, C26.1, C26.8, C26.9)  (C48.0, C48.1, C48.2, C48.8) |
| Genitourinary | Penis, prostate, testis, unspecified male genital organs, kidney, renal pelvis, ureter, bladder, other and unspecified urinary organs | C60, C61, C62, C63, C64, C65, C66, C67, C68  (C60.0, C60.1, C60.2, C60.8, C60.9)  (C62.0, C62.1, C62.9)  (C63.0, C63.1, C63.2, C63.7, C63.8, C63.9)  (C67.0 – C67.9)  (C68.0, C68.1, C68.8, C68.9) |
| Gynaecological | Vulva, vagina, cervix uteri, corpus uteri, uterus, ovary, other unspecific female genital organs, placenta | C51, C52, C53, C54, C55, C56, C57, C58  (C51.0, C51.1, C51.2, C51.8, C51.9)  (C53.0, C53.1, C53.8, C53.9)  (C54.0, C54.1, C54.2, C54.3, C54.8, C54.9)  (C57.0 – C57.9 excl C57.5, C57.6) |
| Haematological | Hodgkin lymphoma, follicular lymphoma, non-follicular lymphoma, mature T/NK-cell lymphomas, other and unspecified types of non-Hodgkin lymphoma, other and unspecified types of T/NK-cell lymphomas, malignant immunoproliferative diseases, multiple myeloma, malignant plasma cell neoplasms, lymphoid leukaemia, myeloid leukaemia, monocytic leukaemia, other leukaemias of specified cell type, leukaemia of unspecified cell type, other and unspecified malignant neoplasms of lymphoid, haematopoietic and related tissue | C81, C82, C83, C84, C85, C86, C87, C88, C90, C91, C92, C93, C94, C95, C96  (C81.0 – C81.9 excl C81.5, C81.6, C81.8)  (C82.0 – C82.9, excl C82.8)  (C83.0, C83.1, C83.3, C83.5,C83.7,C83.8, C83.9)  (C84.0 – C84.9, excl C84.2, C84.3)  (C85.1, C85.2, C85.7, C85.9)  (C86.0 – C86.6)  (C88.0, 88.2, 88.3, 88.4, 88.7, 88.9)  (C90.0 – C90.3)  (C91.0 – C91.9 excl C91.2)  (C92.0 – C92.9)  (C93.0, C93.1, C93.3, C93.7, C93.9)  (C94.0, C94.2, C94.3, C94.4, C94.6, C94.7)  (C95.0, C95.1, C95.7, C95.9)  (C96.0 – C96.9 excl C96.1, C96.3) |
| Head and neck | Lip, base of tongue, other and unspecified parts of tongue, gum, floor of mouth, palate, other and unspecified parts of mouth, parotid gland, other and unspecified major salivary glands, tonsil, orophanynx, nasopharynx, piriform sinus, hypopharynx, other and ill-defined sites in the lip, oral cavity and pharynx, nasal cavity and middle ear, accessory sinuses, larynx, trachea | C00, C01, C02, C03, C04, C05, C06, C07, C08, C09, C10, C11, C12, C13, C14, C30, C31, C32, C33  (C00.0 – C00.9 excl C00.7)  (C02.0 – C02.9 excl C02.5, C02.6, C02.7)  (C03.0, C03.1, C03.9)  (C04.0, C04.1, C04.8, C04.9)  (C05.0, C05.1, C05.2, C05.8, C05.9)  (C06.0, C06.1, C06.2, C06.8, C06.9)  (C08.0, C08.1, C08.8, C08.9)  (C09.0, C09.1, C09.8, C09.9)  (C10. 0 – C10.9 excl C10.5, C10.6, C10.7)  (C11.0, C11.1, C11.2, ,C11.3, C11.8, C11.9)  (C13.0, C13.1, C13.2, C13.8, C13.9)  (C14.0, C14.2, C14.8)  (C30.0, C30.1)  (C31.0, C31.1, C31.2, C31.3, C31.8, C31.9)  (C32.0, C32.1, C32.2, C32.3, C32.8, C32.9) |
| Lung and other thoracic | Mesothelioma, bronchus and lung, thymus, mediastinum and pleura, other and ill-defined sites in the respiratory system and intrathoracic organs | C45, C34, C37, C38, C39  (C45.0, C45.1, C45.2, C45.7, C45.9)  (C34.0, C34.1, C34.2, C34.3, C34.8, C34.9)  (C38.1, C38.2, C38.3, C38.4, C38.8)  (C39.0, C39.8, C39.9) |
| Melanoma | Melanoma | C43  (C43.0 – C43.9) |
| Unknown primary | Unknown primary | C76, C77, C78, C79, C80  (C76.0 – C76.8, excl C76.6)  (C77.0 – C77.9, excl C77.6, C77.7)  (C78.0 – C78.8)  (C79.0 – C79.9)  (C80.0, C80.9) |
| Abbreviations: ICD, International Classification of Disease | | |

| **Table S2** Food groups and respective food items for the generation of dietary patterns | | | |
| --- | --- | --- | --- |
|  |  | **Food groups** | **Food items** |
| **Cereals**  (5 groups) | 1 | White pasta, rice, and other cereals | Pasta, rice, couscous, sushi, and pasta-based soups |
|  | 2 | Wholemeal pasta, rice, and other cereals | Pasta, rice, and other grains (i.e., bulgur wheat, millet pearl barley) |
|  | 3 | Breads, white and wholemeal | Sliced bread, baguette, bap, rolls, garlic bread, naan, and other breads (i.e., crumpets, tortilla wraps, breadsticks) |
|  | 4 | High-fibre breakfast cereals | Porridge, muesli, and cereals (plain, bran, wholewheat) |
|  | 5 | Other breakfast cereals | Sweetened and other cereals |
| **Milk and milk products**  (4 groups) | 6 | Milks, whole and skimmed | Cow and soya milks |
|  | 7 | Dairy beverages | Dairy-based smoothie, yoghurt drinks, milkshakes, flavoured milk,  cappuccino, latte, and hot chocolates |
|  | 8 | Cheese | Hard cheese, soft cheese, blue cheese, mozzarella, goat cheese, spreadable cheese, cottage cheese, fetta cheese and other cheeses |
|  | 9 | Yoghurt, full and low fat | Plain and flavoured yoghurts |
| **Eggs and egg** **dishes**  (1 group) | 10 | Eggs and eggs dishes | Whole egg, omelette, egg in sandwiches, scotch egg, and other egg dishes |
| **Fats and oils**  (3 groups) | 11 | Butter and lard, spreading and cooking | Butter and lard |
|  | 12 | Spreads and oils, not polyunsaturated, spreading and cooking | Olive-based spread, hard margarine,  soya/vegan/other milk-free margarine, vegetable oil, olive oil  rapeseed oil, and other oils |
|  | 13 | Spreads and oils,  Polyunsaturated, spreading and cooking | Polyunsaturated margarine and  sunflower oils |
| **Meat and meat products**  (6 groups) | 14 | Processed meats | Bacon, ham, and sausages |
|  | 15 | Beef, veal, lamb, pork and dishes | Beef, lamb or mutton or pork (i.e., roast, steak, mince, curry, burger, chops, stew), and meat- or poultry-based soups |
|  | 16 | Coated chicken and turkey | Breaded or deep-fried chicken or turkey |
|  | 17 | Chicken and turkey dishes | Chicken and turkey (i.e., roasts, drumsticks and curry) |
|  | 18 | Offal products and dishes | Liver, liver pate, and other meats (i.e., duck, goose, kidney) |
|  | 19 | Meat alternatives | Vegetarian sausages or burgers, tofu, tempeh, textured vegetable protein, soya mince, Quorn and other vegetarian alternatives (i.e., nut roast) |
| **Fish and fish dishes**  (4 groups) | 20 | White fish | White fish (i.e., cod, haddock, fish pie) and fish-based soups |
|  | 21 | White fish, breaded or battered | Battered or breaded fish |
|  | 22 | Other fish, shellfish, and fish dishes | Prawns, lobster or crab, shellfish, and other fish |
|  | 23 | Oily fish | Oily fish (i.e., salmon, tinned salmon, herring, mackerel, sardines or tuna steak) and tinned tuna |
| **Vegetables and potatoes**  (4 groups) | 24 | Vegetables, raw and cooked | Mixed vegetables or vegetable pieces, coleslaw, mixed side salad, beetroot, broccoli, butternut squash, carrot, cauliflower, celery, courgette, cucumber, garlic, leek, lettuce, onion, parsnip, potatoes, sweet pepper, sprouts, sweet corn, sweet potato, tomato, turnip/swede, watercress  other vegetables (i.e., celeriac, asparagus, fennel, aubergine, pumpkin), and vegetables-based soups |
|  | 25 | Potatoes | Potatoes (i.e., fried, chips, roasted) |
|  | 26 | Vitamin D rich vegetables | Mushroom, cabbage or kale, and spinach |
|  | 27 | Beans, lentils, and pulses | Baked beans, lentils, broad beans, other beans, peas, and lentil-based soups |
| **Nuts and seeds**  (1 group) | 28 | Nuts and seeds | Peanuts, peanut butter, roasted and salted nuts (i.e., almonds, cashews, pistachios), unsalted nuts (i.e., almonds, cashews, walnuts), and seeds (i.e., sunflower, pumpkin, linseeds) |
| **Fruit**  (1 group) | 29 | Fruits, including dried fruit | Stewed or cooked fruit, dried fruit, prunes, mixed fruit, apple, banana, berries, cherry, grapefruit, grape, mango, melon, orange, orange-like fruit (i.e., satsuma, clementine, mandarin), peach or nectarine, pear, pineapple, plum, other fruit (i.e., pomegranate, kiwi, papaya), and  avocados |
| **Discretionary foods**  (5 groups) | 30 | Crisps and savoury snacks | Crisps, crackers or crispbreads, cheesy biscuits, oat cakes, olives,  Yorkshire pudding, Indian snacks (i.e., pakora, onion-bhajis or samosas), pizza, and snack pots |
|  | 31 | Biscuits, buns, cakes, pastries, fruit pies, and puddings | Crumble topping, pancakes or crepes, croissants, Danish pastry, scones or cobbler, fruit cake, cake, doughnut, sponge pudding, cheesecake, sweet biscuits, and cereal bars |
|  | 32 | Dairy and soy desserts | Ice-cream, custard, and other milk-based desserts (*i.e., mousse, tiramisu, crème caramel)* |
|  | 33 | Sauces and spreads | Jam or honey, cream, yeast extract, hummus, chutney or pickles, tomato ketchup, brown sauce, mayonnaise, pesto, tomato-based sauces, cheese sauce, whit sauce, gravy, and other sauces |
|  | 34 | Sugar and chocolate confectionary | Chocolate bars, chocolate (white, milk or plain dark), chocolate or yoghurt covered raisins, and chocolate covered biscuits |
| **Non-alcoholic beverages**  (4 groups) | 35 | Fruit juice | Fruit smoothie, orange, grapefruit, and other fruit or vegetable juices |
|  | 36 | Soft drinks, not diet | Carbonated drinks |
|  | 37 | Soft drinks, diet | Low calorie or diet carbonated drinks |
|  | 38 | Tea and coffee | Instant, filtered, americano or cafetiere coffee, espresso, other coffee drinks, standard or green tea, rooibos or red bush tea, herbal or fruit tea, and other tea or infusions |
| **Alcoholic beverages**  (3 groups) | 39 | Spirits and liqueurs | Spirits and other alcoholic drinks |
|  | 40 | Wine | Red, rose or white wine, sherry, fortified wine, and port |
|  | 41 | Beer and cider | Beer, larger, and cider |

| **Table S3** Baseline characteristics of included versus excluded participants with cancer, overall and by sex | | | | | | |
| --- | --- | --- | --- | --- | --- | --- |
| **Characteristics** | **Included (n = 2,415)** | | | **Excluded (n = 49,102)** | | |
|  | **Total** | **Male** | **Female** | **Total** | **Male** | **Female** |
| **N (%)** | 2,415 (100.0) | 950 (39.3) | 1,465 (60.7) | 49,102 (100.0) | 22,271 (45.4) | 26,831 (54.6) |
| **Age (years),** mean ± SD | 59.7 ± 7.1 | 61.3 ± 6.7 | 58.7 ± 7.1 | 60.1 ± 6.9 | 61.3 ± 6.3 | 59.0 ± 7.2 |
| **Cancer type,** n (%) |  |  |  |  |  |  |
| Bone and soft tissue | 19 (0.8) | 15 (1.6) | 4 (0.3) | 377 (0.8) | 229 (1.0) | 148 (0.6) |
| Breast | 847 (35.1) | 8 (0.8) | 839 (57.3) | 13,256 (27.0) | 67 (0.3) | 13,189 (49.2) |
| Central or peripheral nervous system | 25 (1.0) | 13 (1.4) | 12 (0.8) | 739 (1.5) | 418 (1.9) | 321 (1.2) |
| Endocrine and thyroid | 43 (1.8) | 10 (1.1) | 33 (2.3) | 564 (1.2) | 139 (0.6) | 425 (1.6) |
| Gastrointestinal | 285 (11.8) | 165 (17.4) | 120 (8.2) | 7,885 (16.1) | 4,584 (20.6) | 3,301 (12.3) |
| Genitourinary | 523 (21.7) | 497 (52.3) | 26 (1.8) | 11,398 (23.2) | 10,692 (48.0) | 700 (2.6) |
| Gynaecological | 183 (7.6) | N/A | 183 (12.5) | 3,461 (7.1) | N/A | 3,461 (12.9) |
| Haematological | 170 (7.0) | 99 (10.4) | 71 (4.9) | 4,054 (8.3) | 2,270 (10.2) | 1,784 (6.7) |
| Head and neck | 63 (2.6) | 39 (4.1) | 24 (1.6) | 1,303 (2.7) | 900 (4.0) | 403 (1.5) |
| Lung and other thoracic | 23 (1.0) | 14 (1.5) | 9 (0.6) | 2,573 (5.2) | 1,425 (6.4) | 1,148 (4.3) |
| Melanoma | 214 (8.9) | 82 (8.6) | 132 (9.0) | 3,009 (6.1) | 1,314 (5.9) | 1,695 (6.3) |
| Unknown primary | 20 (0.8) | 8 (0.8) | 12 (0.8) | 489 (1.0) | 233 (1.1) | 256 (1.0) |
| **Years since cancer diagnosis,** mean ± SD^a^ | 7.1 ± 6.3 | 5.9 ± 5.9 | 7.8 ± 6.5 | N/A | N/A | N/A |
| **Ethnic background,** n (%) |  |  |  |  |  |  |
| Prefer not to answer | 4 (0.2) | 1 (0.1) | 3 (0.2) | 166 (0.3) | 107 (0.5) | 59 (0.2) |
| Anglo-Saxon (white, British, Irish) | 2,337 (96.8) | 923 (97.2) | 1,414 (96.5) | 47,144 (96.2) | 21,371 (96.1) | 25,773 (96.2) |
| Other | 74 (3.1) | 26 (2.7) | 48 (3.3) | 1,716 (3.5) | 753 (3.4) | 963 (3.6) |
| **Townsend Deprivation Index,** mean ± SD | -1.4 ± 2.8 | -1.4 ± 2.8 | -1.4 ± 2.8 | -1.3 ± 3.1 | -1.3 ± 3.2 | -1.4 ± 3.0 |
| **Smoking status,** n (%) |  |  |  |  |  |  |
| Never | 1,291 (53.5) | 443 (46.6) | 848 (57.9) | 23,776 (48.5) | 9,092 (40.9) | 14,684 (54.8) |
| Previous | 958 (39.7) | 440 (46.3) | 518 (35.4) | 19,410 (39.6) | 10,049 (45.2) | 9,361 (34.9) |
| Current | 166 (6.9) | 67 (7.1) | 99 (6.8) | 5,564 (11.3) | 2,953 (13.4) | 2,611 (9.7) |
| **IPAQ physical activity group,** n (%)^b^ |  |  |  |  |  |  |
| Low | 425 (17.6) | 167 (17.6) | 258 (17.6) | 7,788 (20.4) | 3,669 (20.2) | 4,119 (20.7) |
| Moderate | 1,043 (43.2) | 388 (40.8) | 655 (44.7) | 15,754 (41.3) | 7,158 (39.3) | 8,596 (43.2) |
| High | 947 (39.2) | 395 (41.6) | 552 (37.7) | 14,571 (38.2) | 7,381 (40.5) | 7,190 (36.1) |
| **BMI (kg/m^2^),** mean ± SD | 27.1 ± 4.6 | 27.6 ± 3.9 | 26.8 ± 4.9 | 27.7 ± 4.9 | 27.9 ± 4.2 | 27.5 ± 5.3 |
| **Overweight (BMI),** n (%)^c^ | 1,584 (65.6) | 707 (74.4) | 877 (59.9) | 34,157 (69.6) | 16,925 (76.0) | 17,232 (64.2) |
| **Obese (BMI),** n (%)^c^ | 540 (22.4) | 234 (24.6) | 306 (20.9) | 13,163 (26.8) | 6,010 (27.0) | 7,153 (26.7) |
| **Energy intake (kJ/day),** mean ± SD | 8477 ± 2616 | 9457 ± 2850 | 7842 ± 2235 | 8941 ± 3450 | 9675 ± 3541 | 8299 ± 3235 |
| **Malnutrition,** n (%)^d^ |  |  |  |  |  |  |
| Well nourished | 1,987 (82.3) | 772 (81.3) | 1,215 (82.9) | 42,399 (86.4) | 19,365 (87.0) | 23,034 (85.9) |
| Malnourished | 428 (17.7) | 178 (18.7) | 250 (17.1) | 6,703 (13.7) | 2,906 (13.1) | 3,797 (14.2) |
| Mild-moderate | 357 (14.8) | 141 (14.8) | 216 (14.7) | 5,305 (10.8) | 2,118 (9.5) | 3,187 (11.9) |
| Severe | 71 (2.9) | 37 (3.9) | 34 (2.3) | 1,398 (2.8) | 788 (3.5) | 610 (2.3) |
| **Low muscle mass,** n (%)^e^ |  |  |  |  |  |  |
| Normal muscle mass | 2,295 (95.0) | 873 (91.9) | 1,422 (97.1) | 44,096 (92.0) | 19,005 (87.7) | 25,091 (95.7) |
| Low muscle mass | 120 (5.0) | 77 (8.1) | 43 (2.9) | 3,818 (8.0) | 2,679 (12.4) | 1,139 (4.3) |
| **Sarcopenia,** n (%)^f^ |  |  |  |  |  |  |
| Non-sarcopenic | 2,258 (93.5) | 890 (93.7) | 1,368 (93.4) | 44,614 (93.6) | 20,349 (94.4) | 24,265 (93.0) |
| Sarcopenic | 157 (6.5) | 60 (6.3) | 97 (6.6) | 3,051 (6.4) | 1,217 (5.6) | 1,834 (7.0) |
| Probable sarcopenia | 142 (5.9) | 46 (4.8) | 96 (6.6) | 2,512 (5.3) | 856 (4.0) | 1,656 (6.4) |
| Sarcopenia | 13 (0.5) | 12 (1.3) | 1 (0.1) | 295 (0.6) | 214 (1.0) | 81 (0.3) |
| Severe sarcopenia | 2 (0.1) | 2 (0.2) | 0 (0.0) | 244 (0.5) | 147 (0.7) | 97 (0.4) |
| Data are reported as n (%) and mean ±SD. Abbreviations: ALST, appendicular lean soft tissue; BMI, body mass index; IPAQ, International Physical Activity Questionnaire.  ^a^Years since diagnosis: number of years from date of first cancer diagnosis to date of baseline assessment visit. Not applicable for ‘excluded’ sample as participants may be diagnosed with cancer following the baseline assessment visit.  ^b^IPAQ physical activity group: ‘low physical activity group’, ≤ 918 MET minute/week; ‘high physical activity group’, > 3706 –1 9,278 MET minute/week.  ^c^Overweight and obese: classified as BMI ≥ 25 kg/m^2^ and BMI ≥ 30 kg/m^2^, respectively.  ^d^Malnutrition: amended definition according to the Global Leadership initiative on Malnutrition criteria. Severe malnutrition defined as malnourishment with BMI < 18.5 kg/m^2^ (< 70 years) and < 20 kg/m^2^ ( ≥ 70 years) or ALST/BMI < 0.84 (male) and < 0.55 (female).  ^e^Low muscle mass: ALST/BMI < 0.84 (males) and < 0.55 (females).  ^f^Sarcopenia: amended definition according to European Working Group on Sarcopenia in Older People 2019 definitions. Probable sarcopenia, handgrip strength < 27 kg (male) and < 16 kg (female); sarcopenia, low handgrip strength plus ALST/BMI < 0.84 (male) and < 0.55 (female); sever sarcopenia, low handgrip strength plus low muscle mass plus low physical performance (slow walking speed).  Number of participants in the ‘excluded’ sample varies due to missing data for one or more demographic variables: Ethnic background and smoking status, n = 49,026; Townsend Deprivation Index, n = 49,056; BMI, n = 48,807; IPAQ physical activity group, n = 38,113; muscle mass, n = 47,914 and; sarcopenia, n = 47,665. | | | | | | |

| **Table S4** Explained variation (%) in food intakes and nutrient response variables for each RRR derived dietary pattern and correlation coefficient between dietary patterns and nutrient response variables in UK Biobank participants with a previous cancer diagnosis and recent cancer diagnosis | | | | | | | | | | |
| --- | --- | --- | --- | --- | --- | --- | --- | --- | --- | --- |
|  | **Explained variation (%)** | | | | | | | **Correlation coefficients** | | |
|  |  |  | | **Nutrient response variables** | | | |  | | |
| **Dietary patterns** | **Food intakes (total)** | | **Nutrient response variables (total)** | **Protein (g/kg/day)** | **PUFA (g/day)** | **Vitamin D (µg/day)** | | **Protein (g/kg/day)** | **PUFA**  **(g/day)** | **Vitamin D (µg/day)** |
| **Previous diagnosis**  **(n = 2,415)** |  | |  |  |  | |  |  |  |  |
| High oily fish and nuts | 2.5 | | 44.0 | 38.5 | 44.2 | | 49.3 | 0.54 | 0.58 | 0.61 |
| Low oily fish | 2.6 | | 19.6 | 2.6 | 21.9 | | 34.2 | 0.21 | 0.61 | -0.76 |
| Meat and dairy | 2.3 | | 11.4 | 22.8 | 10.0 | | 1.5 | 0.82 | -0.54 | -0.21 |
| **Recent diagnosis**  **(n = 491)** |  | |  |  |  | |  |  |  |  |
| High oily fish and nuts – sub | 2.7 | | 43.5 | 31.6 | 45.9 | | 53.1 | 0.49 | 0.59 | 0.64 |
| Low oily fish – sub | 2.5 | | 18.6 | 6.2 | 16.9 | | 32.9 | 0.33 | 0.55 | -0.77 |
| Meat and dairy – sub | 2.4 | | 13.4 | 26.1 | 14.0 | | 0.2 | 0.80 | -0.59 | -0.07 |
| Abbreviations: PUFA, polyunsaturated fatty acids; RRR, reduced rank regression. Explained variation (%) represents the unique variation in the input variables (food intakes) and dependent variable (nutrient response variables i.e., protein, PUFA and vitamin D) that was predictable from the extracted dietary patterns. | | | | | | | | | | |

| **Table S5** Factor loadings of the 41 food groups derived from UK Biobank participants with a previous cancer diagnosis and recent cancer diagnosis, by dietary pattern | | | | | | |
| --- | --- | --- | --- | --- | --- | --- |
|  | **Previous diagnosis (n = 2,415)** | | | **Recent diagnosis (n = 491)** | | |
| **Food groups** | **High oily fish and nuts** | **Low oily fish** | **Meat and dairy** | **High oily fish and potatoes** | **Low oily fish and high potatoes** | **High meat and low potatoes** |
| White pasta, rice, and cereals | -0.05 | 0.02 | 0.21 | -0.08 | 0.06 | 0.27 |
| Wholemeal pasta, rice, and cereals | -0.01 | 0.00 | 0.11 | 0.02 | 0.05 | 0.15 |
| Breads | 0.18 | 0.19 | 0.04 | 0.14 | 0.16 | 0.15 |
| High-fibre breakfast cereals | 0.09 | 0.08 | 0.09 | 0.14 | 0.10 | 0.04 |
| Other breakfast cereals | 0.04 | 0.03 | 0.06 | -0.01 | 0.08 | 0.08 |
| Milk | 0.10 | 0.02 | 0.18 | 0.12 | 0.01 | 0.17 |
| Dairy beverages | 0.05 | 0.02 | 0.18 | 0.03 | 0.05 | 0.14 |
| Cheeses | 0.10 | 0.07 | 0.20 | 0.07 | 0.10 | 0.22 |
| Yoghurt, low and full fat | 0.06 | -0.06 | 0.18 | 0.07 | -0.05 | 0.17 |
| Eggs and eggs dishes | 0.22 | -0.08 | -0.03 | 0.18 | -0.07 | 0.02 |
| Butter and lard | -0.00 | -0.03 | 0.05 | 0.07 | -0.07 | 0.07 |
| Spreads and oils, not polyunsaturated | 0.15 | 0.07 | -0.03 | 0.12 | 0.06 | -0.02 |
| Spreads and oils, polyunsaturated | 0.13 | 0.22 | -0.18 | 0.15 | 0.19 | -0.20 |
| Processed meats | 0.06 | 0.08 | -0.03 | 0.15 | 0.09 | -0.04 |
| Beef, veal, lamb, pork and dishes | 0.10 | 0.08 | 0.50 | 0.05 | 0.12 | 0.48 |
| Coated chicken and turkey | 0.03 | 0.08 | -0.08 | 0.05 | 0.09 | -0.08 |
| Chicken and turkey dishes | 0.15 | 0.16 | 0.31 | 0.19 | 0.25 | 0.25 |
| Offal products and dishes | -0.01 | 0.03 | 0.02 | 0.00 | 0.03 | 0.01 |
| Meat alternatives | 0.02 | 0.12 | 0.00 | -0.01 | 0.05 | 0.04 |
| White fish | -0.04 | 0.05 | 0.10 | -0.06 | 0.10 | 0.05 |
| White fish, breaded or battered | 0.02 | 0.18 | -0.13 | 0.00 | 0.06 | -0.08 |
| Other fish, shellfish, and fish dishes | 0.06 | -0.08 | -0.03 | 0.07 | -0.09 | 0.01 |
| Oily fish | 0.66 | -0.63 | -0.18 | 0.65 | -0.63 | -0.09 |
| Vegetables, raw and cooked | 0.21 | 0.01 | 0.20 | 0.15 | 0.07 | 0.16 |
| Potatoes | 0.31 | 0.48 | -0.41 | 0.33 | 0.41 | -0.48 |
| Vitamin D rich vegetables | 0.08 | 0.02 | 0.11 | 0.14 | 0.03 | 0.01 |
| Beans, lentils, and pulses | 0.09 | 0.10 | 0.11 | 0.03 | 0.15 | 0.09 |
| Nuts and seeds | 0.32 | 0.23 | -0.15 | 0.27 | 0.25 | -0.25 |
| Fruit, included dried | 0.10 | -0.01 | 0.16 | 0.09 | -0.04 | 0.15 |
| Crisps and savoury snacks | 0.04 | 0.12 | -0.02 | 0.04 | 0.14 | -0.07 |
| Biscuits, buns, cakes, pastries, fruit pies, and puddings | 0.15 | 0.15 | -0.12 | 0.17 | 0.16 | -0.09 |
| Dairy and soy desserts | 0.02 | 0.07 | 0.03 | -0.01 | 0.10 | 0.03 |
| Sauces and spreads | 0.22 | 0.16 | -0.03 | 0.21 | 0.16 | 0.05 |
| Sugar and chocolate confectionary | 0.07 | 0.10 | -0.03 | 0.03 | 0.08 | -0.06 |
| Fruit juice | 0.05 | -0.04 | 0.09 | 0.06 | -0.06 | -0.00 |
| Soft drinks, not diet | 0.01 | -0.01 | -0.07 | -0.08 | -0.05 | -0.08 |
| Soft drinks, diet | -0.03 | -0.02 | -0.00 | -0.07 | -0.09 | -0.00 |
| Tea and coffee | 0.13 | 0.04 | 0.15 | 0.15 | 0.02 | 0.09 |
| Spirits and liqueurs | 0.00 | -0.02 | -0.04 | 0.05 | -0.03 | 0.00 |
| Wine | 0.02 | -0.04 | 0.02 | 0.07 | -0.08 | 0.00 |
| Beer and cider | 0.03 | 0.01 | -0.09 | -0.00 | -0.08 | -0.03 |
|  | | | | | | |

| **Table S6** Response variable, energy, nutrient and key food group intakes across tertiles of dietary pattern scores in UK Biobank participants with a recent cancer diagnosis (n = 491) | | | | | | |
| --- | --- | --- | --- | --- | --- | --- |
|  | **Tertiles of dietary pattern score** | | | | | |
|  | **Tertile 1**  (n = 164) | | **Tertile 2**  (n = 164) | | **Tertile 3**  (n = 163) | |
|  | Mean ± SD | Median (IQR) | Mean ± SD | Median (IQR) | Mean ± SD | Median (IQR) |
| ***‘High oily fish and nuts - sub’ pattern*** | | | | | | |
| ***Response variables*** |  |  |  |  |  |  |
| Protein (g/kg/day) | 0.8 ± 0.3 | 0.8 (0.6, 1.0) | 1.1 ± 0.3 | 1.1 (0.9, 1.3) | 1.3 ± 0.4 | 1.3 (1.0, 1.6) |
| PUFA (g/day) | 8.2 ± 4.9 | 6.9 (4.7, 10.9) | 13.2 ± 5.6 | 12.4 (8.9, 16.9) | 19.9 ± 9.0 | 18.2 (13.1, 25.7) |
| Vitamin D (µg/day) | 1.2 ± 1.0 | 1.0 (0.4, 1.7) | 2.0 ± 1.2 | 1.8 (1.1, 2.7) | 5.9 ± 5.0 | 3.9 (2.0, 9.4) |
| **Foods (g/day)** |  |  |  |  |  |  |
| Oily fish (+) | 0 ± 0 | 0 (0, 0) | 2.2 ± 11.9 | 0 (0, 0) | 52.9 ± 67.7 | 0 (0, 114) |
| Potatoes (+) | 2.9 ± 17.2 | 0 (0, 0) | 11.5 ± 35.8 | 0 (0, 0) | 42.2 ± 82.0 | 0 (0, 68.8) |
| Nuts and seeds (+) | 1.5 ± 6.5 | 0 (0, 0) | 4.2 ± 13.2 | 0 (0, 0) | 11.5 ± 25.1 | 0 (0, 0) |
| Sauces and spreads (+) | 11.5 ± 15.6 | 0 (0, 32.5) | 19.4 ± 16.0 | 32.5 (0, 32.5) | 20.1 ± 15.8 | 32.5 (0, 32.5) |
| Chicken, turkey and dishes (+) | 16.7 ± 44.7 | 0 (0, 0) | 37.6 ± 64.5 | 0 (0, 76.2) | 43.5 ± 78.5 | 0 (0, 152.3) |
| Eggs and dishes (+) | 8.6 ± 25.7 | 0 (0, 0) | 25.2 ± 52.8 | 0 (0, 53.0) | 36.6 ± 67.1 | 0 (0, 53.0) |
| Biscuits, buns, and cakes (+) | 24.2 ± 44.1 | 0 (0, 30.0) | 42.9 ± 64.2 | 0 (0, 65.0) | 55.7 ± 83.0 | 10 (0, 110.0) |
| Processed meats (+) | 9.1 ± 21.7 | 0 (0, 0) | 12.2 ± 27.2 | 0 (0, 5.8) | 23.0 ± 43.1 | 0 (0, 28.3) |
| Spreads and oils, PUFA (+) | 3.0 ± 7.6 | 0 (0, 0) | 5.3 ± 12.0 | 0 (0, 0) | 7.1 ± 14.8 | 0 (0, 0) |
| Vegetables (+) | 219.4 ± 203.9 | 184.4 (0, 367.8) | 263.2 ± 238.4 | 211.3 (55.0, 396.3) | 295.1 ± 251.4 | 260.0 (90.0, 420.0) |
| ***Nutrients*** |  |  |  |  |  |  |
| Total energy (kJ/day) | 6903 ± 2278 | 6735 (5457, 7815) | 8803 ± 2061 | 8448 (7411, 10147) | 10506 ± 2624 | 10251 (8534, 12140) |
| Protein (% energy) | 14.9 ± 4.2 | 14.1 (11.9, 17.1) | 17.0 ± 4.3 | 16.5 (14.0, 19.6) | 17.1 ± 4.1 | 16.6 (14.2, 18.6) |
| Total fat (% energy) | 29.1 ± 8.1 | 29.8 (24.1, 33.5) | 32.8 ± 7.6 | 32.4 (28.1, 37.4) | 35.5 ± 7.5 | 35.6 (30.3, 40.6) |
| Saturated fat (% energy) | 11.9 ± 4.3 | 11.7 (8.6, 14.9) | 12.7 ± 4.0 | 12.9 (9.9, 15.2) | 12.6 ± 4.1 | 12.3 (9.9, 15.1) |
| Carbohydrate (% energy) | 54.1 ± 10.2 | 55.0 (47.7, 60.8) | 50.3 ± 9.3 | 50.0 (44.3, 57.0) | 46.6 ± 9.5 | 48.0 (39.4, 53.5) |
| Total sugars (% energy) | 27.2 ± 9.2 | 27.2 (20.7, 32.4) | 24.5 ± 7.8 | 24.4 (19.7, 30.0) | 22.4 ± 7.4 | 22.8 (17.4, 27.0) |
| Dietary fibre (g/day) | 13.9 ± 5.9 | 13.8 (9.0, 18.4) | 16.9 ± 6.4 | 16.6 (12.7, 20.8) | 19.8 ± 8.1 | 18.9 (14.7, 23.5) |
| ***’Low oily fish – sub’ pattern*** | | | | | | |
| ***Response variables*** |  |  |  |  |  |  |
| Protein (g/kg/day) | 0.9 ± 0.4 | 0.9 (0.7, 1.1) | 1.0 ± 0.3 | 1.0 (0.8, 1.2) | 1.3 ± 0.4 | 1.2 (1.0, 1.5) |
| PUFA (g/day) | 11.0 ± 6.9 | 9.6 (5.5, 14.8) | 10.7 ± 5.2 | 9.8 (6.9, 13.1) | 19.7 ± 8.8 | 18.1 (13.8, 24.6) |
| Vitamin D (µg/day) | 5.1 ± 5.1 | 3.2 (1.1, 8.9) | 1.9 ± 1.8 | 1.4 (0.8, 2.7) | 2.0 ± 2.0 | 1.6 (0.9, 2.4) |
| **Foods (g/day)** |  |  |  |  |  |  |
| Oily fish (-) | 48.2 ± 66.6 | 0 (0, 114.0) | 2.0 ± 14.7 | 0 (0, 0) | 4.6 ± 22.0 | 0 (0, 0) |
| Potatoes (+) | 4.0 ± 22.0 | 0 (0, 0) | 4.4 ± 21.3 | 0 (0, 0) | 48.3 ± 83.2 | 0 (0, 137.5) |
| Chicken, turkey and dishes (+) | 14.9 ± 46.9 | 0 (0, 0) | 24.6 ± 53.3 | 0 (0, 0) | 58.4 ± 81.4 | 0 (0, 152.3) |
| Nuts and seeds (+) | 2.4 ± 10.9 | 0 (0, 0) | 2.3 ± 8.2 | 0 (0, 0) | 12.5 ± 25.4 | 0 (0, 0) |
| Spreads and oils, PUFA (+) | 2.9 ± 9.6 | 0 (0, 0) | 3.2 ± 8.0 | 0 (0, 0) | 9.3 ± 15.7 | 0 (0, 20.0) |
| Breads (+) | 79.9 ± 72.7 | 72.0 (0, 120.0) | 93.3 ± 77.0 | 72.0 (56.7, 120.0) | 127.1 ± 133.1 | 108.0 (72.0, 144.0) |
| Sauces and spreads (+) | 12.5 ± 15.9 | 0 (0, 32.5) | 17.2 ± 16.3 | 32.5 (0, 32.5) | 21.3 ± 15.5 | 32.5 (0, 32.5) |
| Biscuits, buns, and cakes (+) | 26.9 ± 47.5 | 0 (0, 30.0) | 38.5 ± 59.5 | 0 (0, 55.0) | 57.5 ± 48.9 | 10 (0, 110.0) |
| Beans, lentils, and pulses (+) | 27.6 ± 58.7 | 0 (0, 26.3) | 39.0 ± 62.7 | 0 (0, 70.0) | 47.4 ± 70.7 | 0 (0, 90.0) |
| Crisps and savoury snacks (+) | 18.9 ± 92.1 | 0 (0, 20.0) | 27.9 ± 85.5 | 0 (0, 38.7) | 52.4 ± 196.8 | 0 (0, 40.0) |
| ***Nutrients*** |  |  |  |  |  |  |
| Total energy (kJ/day) | 7809 ± 2657 | 7606 (5934, 9506) | 8134 ± 2313 | 7592 (6709) | 10248 ± 2635 | 10038 (8221, 12092) |
| Protein (% energy) | 15.8 ± 4.7 | 15.2 (12.5, 18.0) | 16.9 ± 4.5 | 16.5 (13.6, 19.4) | 16.3 ± 3.7 | 16.1 (14.0, 18.0) |
| Total fat (% energy) | 31.4 ± 9.2 | 31.0 (25.3, 37.2) | 31.7 ± 7.4 | 31.3 (27.0, 36.2) | 34.3 ± 7.4 | 34.3 (29.1, 39.0) |
| Saturated fat (% energy) | 12.1 ± 4.4 | 11.5 (8.6, 15.2) | 12.9 ± 4.1 | 13.1 (10.1, 15.7) | 12.2 ± 3.8 | 12.1 (9.5, 14.6) |
| Carbohydrate (% energy) | 50.1 ± 12.2 | 50.6 (41.7, 58.6) | 51.4 ± 9.0 | 51.8 (45.9, 57.6) | 49.5 ± 8.8 | 50.0 (43.3, 56.2) |
| Total sugars (% energy) | 26.0 ± 9.5 | 25.4 (20.1, 30.8) | 25.3 ± 8.0 | 24.8 (20.1, 30.2) | 22.8 ± 7.2 | 22.8 (18.1, 28.0) |
| Dietary fibre (g/day) | 14.5 ± 6.4 | 14.8 (9.5, 19.5) | 15.6 ± 6.1 | 14.8 (10.8, 19.8) | 20.5 ± 7.8 | 18.8 (15.3, 24.4) |
| ***‘Meat and dairy - sub’ pattern*** | | | | | | |
| ***Response variables*** |  |  |  |  |  |  |
| Protein (g/kg/day) | 0.9 ± 0.3 | 0.9 (0.6, 1.0) | 1.0 ± 0.3 | 1.0 (0.8, 1.2) | 1.3 ± 0.4 | 1.3 (1.1, 1.6) |
| PUFA (g/day) | 17.5 ± 10.1 | 16.4 (9.7, 23.9) | 12.2 ± 7.3 | 11.0 (6.6, 16.6) | 11.6 ± 5.1 | 11.3 (7.5, 14.6) |
| Vitamin D (µg/day) | 3.4 ± 4.2 | 1.6 (0.8, 3.2) | 2.8 ± 3.5 | 1.7 (0.9, 3.4) | 2.9 ± 3. 2 | 2.0 (1.1, 3.3) |
| **Foods (g/day)** |  |  |  |  |  |  |
| Beef, veal, lamb and pork (+) | 13.2 ± 43.3 | 0 (0, 0) | 42.4 ± 71.7 | 0 (0, 83.8) | 109.6 ± 107.3 | 163.3 (0, 167.5) |
| Potatoes (-) | 47.8 ± 82.8 | 0 (0, 137.5) | 7.1 ± 28.9 | 0 (0, 0) | 1.5 ± 12.3 | 0 (0, 0) |
| White pasta, rice and cereals (+) | 12.6 ± 51.5 | 0 (0, 0) | 40.8 ± 82.9 | 0 (0, 0) | 67.9 ± 110.3 | 0 (0, 180.0) |
| Chicken, turkey and dishes (+) | 15.3 ± 51.4 | 0 (0, 0) | 31.1 ± 58.9 | 0 (0, 0) | 51.4 ± 77.0 | 0 (0, 152.3) |
| Nuts and seeds (-) | 11.0 ± 23.7 | 0 (0, 0) | 4.0 ± 14.5 | 0 (0, 0) | 2.0 ± 8.8 | 0 (0, 0) |
| Cheeses (+) | 9.5 ± 15.1 | 0 (0, 15.0) | 15.3 ± 21.9 | 0 (0, 30) | 22.0 ± 27.3 | 15 (0, 30) |
| Spreads and oils, PUFA (-) | 7.3 ± 15.2 | 0 (0, 0) | 5.2 ± 10.8 | 0 (0, 0) | 2.9 ± 8.4 | 0 (0, 0) |
| Milk (+) | 142.3 ± 95.8 | 127.5 (82.5, 210.0) | 149.6 ± 129.3 | 137.5 (82.5, 210.0) | 196.4 ± 169.0 | 182.5 (100.0, 237.5) |
| Yoghurt (+) | 45.0 ± 101.2 | 0 (0, 0) | 83.5 ± 115.7 | 0 (0, 250.0) | 92.4 ± 135.8 | 0 (0, 250.0) |
| Vegetables (+) | 218.7 ± 227.4 | 175.0 (0, 350.0) | 266.0 ± 230.2 | 213.1 (85.0, 417.5) | 292.9 ± 238.6 | 260.0 (91.4, 420.0) |
| ***Nutrients*** |  |  |  |  |  |  |
| Total energy (kJ/day) | 8441 ± 2782 | 7934 (6604, 10013) | 8241 ± 2675 | 7976 (6504, 9793) | 9524 ± 2645 | 9452 (7535, 11393) |
| Protein (% energy) | 13.5 ± 2.9 | 13.2 (11.4, 15.3) | 16.5 ± 3.9 | 16.0 (13.7, 18.2) | 19.0 ± 4.2 | 18.1 (16.2, 21.0) |
| Total fat (% energy) | 35.1 ± 8.3 | 35.2 (30.6, 39.9) | 31.3 ± 8.2 | 30.8 (25.9, 37.4) | 31.0 ± 7.3 | 30.3 (26.4, 35.2) |
| Saturated fat (% energy) | 12.3 ± 4.0 | 12.1 (9.0, 15.0) | 12.2 ± 4.2 | 12.0 (9.0, 15.3) | 12.7 ± 4.2 | 12.8 (9.7, 15.1) |
| Carbohydrate (% energy) | 50.5 ± 9.9 | 50.5 (43.6, 58.0) | 50.8 ± 10.2 | 50.7 (44.1, 56.7) | 49.8 ± 10.3 | 51.1 (43.9, 57.4) |
| Total sugars (% energy) | 24.7 ± 8.1 | 24.5 (18.8, 29.6) | 24.9 ± 8.9 | 24.9 (20.0, 30.1) | 24.5 ± 8.2 | 24.2 (19.5, 30.1) |
| Dietary fibre (g/day) | 16.4 ± 6.6 | 15.8 (11.8, 20.4) | 15.9 ± 7.1 | 15.5 (10.7, 19.5) | 18.3 ± 7.8 | 17.1 (13.2, 22.0) |
| Abbreviations: PUFA, polyunsaturated fatty acids; SD, standard deviation.  Non-consumers (n, %): oily fish (415, 84.5); potatoes (424, 86.4); nuts and seeds (427, 87.0); sauces and spreads (234, 47.7); chicken and turkey (384, 78.2); eggs and dishes (374, 76.2); biscuits, buns and cakes (261, 53.2); processed meats (361, 73.5); spreads and oils, PUFA (395, 80.5); vegetables (106, 21.6); bread (92, 18.7); beans and legumes (316, 64.4); crisps and savoury snacks (311, 63.3); beef, veal, lamb and pork (335, 68.2); white pasta and rice (397, 80.9); cheese (271, 55.2); milk (57, 11.6); yoghurt (325, 66.2). | | | | | | |

| **Table S7** Odds Ratios (OR) and 95% confidence intervals (CI) for associations between dietary patterns and prevalent malnutrition, low muscle mass and sarcopenia at the baseline assessment for UK Biobank participants with a previous cancer diagnosis and when adjusted for total energy intake (sensitivity analysis). | | | | | | |
| --- | --- | --- | --- | --- | --- | --- |
|  | **Malnutrition**^1^ | | **Low muscle mass**^2^ | | **Sarcopenia**^3^ | |
| **Dietary Patterns** | **OR (95% CI)** | **P-value** | **OR (95% CI)** | **P-value** | **OR (95% CI)** | **P-value** |
| **Previous diagnosis (n = 2,415)** |  |  |  |  |  |  |
| High oily fish and nuts  Energy adjusted (kJ) | 0.57 (0.50, 0.65)  N/A | < 0.001  N/A | 0.87 (0.72, 1.06)  0.98 (0.77, 1.24) | 0.17  0.85 | 1.10 (0.95, 1.29)  1.12 (0.93, 1.35) | 0.21  0.24 |
| Low oily fish  Energy adjusted (kJ) | 0.81 (0.73, 0.90)  N/A | < 0.001  N/A | 0.93 (0.78, 1.12)  1.00 (0.82, 1.22) | 0.44  0.98 | 1.06 (0.90, 1.24)  1.05 (0.88, 1.25) | 0.52  0.61 |
| Meat and dairy  Energy adjusted (kJ) | 0.94 (0.85, 1.05)  N/A | 0.28  N/A | 0.95 (0.80, 1.14)  0.98 (0.81, 1.18) | 0.61  0.81 | 0.89 (0.75, 1.05)  0.89 (0.75, 1.05) | 0.17  0.15 |
| Abbreviations: CI, confidence interval; OR, odds ratio.  ^1^Malnutrition: amended definition according to the Global Leadership initiative on Malnutrition criteria. Low ALST/BMI for reduced muscle mass criteria. Mild-moderate and severe malnutrition diagnoses were combined.  ^2^Low muscle mass: ALST/BMI.  ^3^Sarcopenia: amended definition according to the European Working Group on Sarcopenia in Older People 2019 definitions. Low ALST/BMI for low muscle mass criteria. Probable, sarcopenia and severe sarcopenia diagnoses combined.  Model adjusted for: age, sex, smoking status, Townsend Deprivation Status, cancer type, and physical activity level and time since diagnosis, or age, sex, smoking status, Townsend Deprivation Status, cancer type, physical activity level, time since diagnosis and energy intake. | | | | | | |

| **Table S8** ORs and 95% confidence intervals for prevalence of components of the GLIM definition of malnutrition at the baseline assessment visit in UK biobank participants with a previous cancer diagnosis and recent cancer diagnosis | | | | | | | | |  |
| --- | --- | --- | --- | --- | --- | --- | --- | --- | --- |
|  | **Phenotypic criteria** | | | | **Etiologic criteria** | | | |  |
|  | **Weight loss^a^** | | **Low BMI^b^** | | **Reduced food intake^c^** | | **Inflammation^d^** | |  |
| **Dietary Patterns** | **OR (95% CI)** | **P-value** | **OR (95% CI)** | **P-value** | **OR (95% CI)** | **P-value** | **OR (95% CI)** | **P-value** |  |
| **Previous diagnosis (n=2,415)** |  |  |  |  |  |  |  |  |  |
| High oily fish and nuts | 1.04 (0.94, 1.15) | 0.48 | 1.16 (0.89, 1.51) | 0.26 | 0.28 (0.24, 0.33) | <0.001 | 0.88 (0.77, 1.01) | 0.06 |  |
| Low oily fish | 0.93 (0.83, 1.03) | 0.15 | 0.97 (0.73, 1.29) | 0.83 | 0.60 (0.54, 0.67) | <0.001 | 1.11 (0.98, 1.04) | 0.10 |  |
| Meat and dairy | 1.00 (0.90, 1.11) | 0.97 | 0.97 (0.73, 1.29) | 0.84 | 0.81 (0.74, 0.89) | <0.001 | 1.00 (0.88, 1.13) | 0.95 |  |
| **Recent diagnosis (n = 491)** |  |  |  |  |  |  |  |  |  |
| High oily fish and nuts - sub | 1.13 (0.90, 1.40) | 0.29 | 1.12 (0.65, 1.92) | 0.70 | 0.27 (0.19, 0.38) | <0.001 | 0.78 (0.59, 1.03) | 0.08 |  |
| Low oily fish - sub | 0.81 (0.65, 1.00) | 0.06 | 1.06 (0.58, 1.94) | 0.85 | 0.59 (0.47, 0.73) | <0.001 | 1.08 (0.83, 1.40) | 0.57 |  |
| Meat and dairy - sub | 1.12 (0.89, 1.41) | 0.89 | 1.28 (0.71, 2.34) | 0.41 | 0.79 (0.64, 0.97) | 0.03 | 0.90 (0.70, 1.15) | 0.40 |  |
| Abbreviations: BMI, body mass index; CI, confidence interval; GLIM, Global Leadership Initiative for Malnutrition; OR, odds ratio.  ^a^Weight loss: self-reported weight loss from 1-year ago.  ^b^Low BMI: < 20 kg/m^2^ if < 70 years or < 22 kg/m^2^ if ≥ 70 years.  ^c^Reduced food intake: < 75% of recommended energy intake, taken from the midpoint of European Society for Clinical Nutrition and Metabolism (ESPEN) guidelines for energy intake in cancer (< 115.5 kj/kg/day).  ^d^Inflammation: CRP > 5 mg/L.  Model adjusted for: age, sex, smoking status, Townsend Deprivation Index, cancer type, physical activity level and time since diagnosis (full sample) or age, sex, smoking status, Townsend Deprivation Index, physical activity level and time since diagnosis (sub-sample). | | | | | | | | | |
